# Realizing asynchronous finite-time robust tracking control of switched flight vehicles by using nonfragile deep reinforcement learning

**DOI:** 10.3389/fnins.2023.1329576

**Published:** 2023-12-21

**Authors:** Haoyu Cheng, Ruijia Song, Haoran Li, Wencheng Wei, Biyu Zheng, Yangwang Fang

**Affiliations:** ^1^Unmanned System Research Institute, Northwestern Polytechnical University, Xi’an, China; ^2^Xi’an Modern Control Technology Research Institute, Xi’an, China; ^3^School of Astronautics, Northwestern Polytechnical University, Xi’an, China

**Keywords:** switched systems, asynchronous switching, deep reinforcement learning, nonfragile control, finite *H*∞ control

## Abstract

In this study, a novel nonfragile deep reinforcement learning (DRL) method was proposed to realize the finite-time control of switched unmanned flight vehicles. Control accuracy, robustness, and intelligence were enhanced in the proposed control scheme by combining conventional robust control and DRL characteristics. In the proposed control strategy, the tracking controller consists of a dynamics-based controller and a learning-based controller. The conventional robust control approach for the nominal system was used for realizing a dynamics-based baseline tracking controller. The learning-based controller based on DRL was developed to compensate model uncertainties and enhance transient control accuracy. The multiple Lyapunov function approach and mode-dependent average dwell time approach were combined to analyze the finite-time stability of flight vehicles with asynchronous switching. The linear matrix inequalities technique was used to determine the solutions of dynamics-based controllers. Online optimization was formulated as a Markov decision process. The adaptive deep deterministic policy gradient algorithm was adopted to improve efficiency and convergence. In this algorithm, the actor–critic structure was used and adaptive hyperparameters were introduced. Unlike the conventional DRL algorithm, nonfragile control theory and adaptive reward function were used in the proposed algorithm to achieve excellent stability and training efficiency. We demonstrated the effectiveness of the presented algorithm through comparative simulations.

## 1 Introduction

Aerospace technology has developed rapidly since the 20th century (Wang et al., 2021; Giacomin and Hemerly, 2022; Wang and Xu, 2022). To satisfy the requirements of scientific exploration, military attack, transportation, industrial assistance, and other domains (Bao et al., 2021), flight vehicle systems are becoming increasingly complex (Wu et al., 2021; Lee and Kim, 2022). As an effective tool for the analysis of complex nonlinear systems, switched systems exhibit considerable potential for use in fast time-variation (Hu et al., 2019), full envelope, structural model mutation (Grigorie et al., 2022), re-modeling (Yue et al., 2019), among others (Chen et al., 2022; Yang et al., 2022).

Switched systems are a critical component of a series of discrete/continuous subsystems, and a switching signal controls the switching logic between these subsystems (Zhang et al., 2019). The switched system exhibits considerable potential for use in theoretical research and engineering applications (Sun and Lei, 2021), such as modeling (Huang et al., 2020), stability analysis (Yang et al., 2020; Zhang and Zhu, 2020), and control problems (Gong et al., 2020; Xiao et al., 2020). The stability analysis of the switched systems is typically used for controller design (Liu et al., 2020). The common Lyapunov function (CLF) method is widely used for stability analysis of arbitrary switching (Jiang et al., 2020). However, ensuring that a CLF is shared by all the subsystems remains challenging. This method is conservative to some degree, which leads to the research is required on the MLF and average dwell time (ADT) methods. Zhao et al. (2012) first studied the stability of the switched systems with ADT switching. In another study, the linear copositive function was extended to the MLF, and the multiple linear copositive Lyapunov function method was used to obtain a sufficient stability criterion for switched systems (Cheng et al., 2017). To obtain tight bounds on the dwell time, the mode-dependent average dwell time (MDADT) method was proposed to overcome the sharing problem of common parameters, and the worst cases were considered in the ADT method. The results were extended to a general case, and the properties of subsystems were considered. Generally, unstable modes may exist during the switching intervals. Therefore, a piecewise multi-Lyapunov function method was proposed in Zhao et al. (2017) for the stability analysis of unstable modes. To avoid dwelling for a long time in subsystems with poor performance and considering the MDADT methods, the slow switching is typically applied to stable modes, and fast switching is applied to unstable modes. Xu et al. (2019) proposed a time-dependent quadratic Lyapunov function method to solve the stability problem with all subsystems unstable. The bounded maximum ADT method is used to obtain the stability conditions of the linear switched system. However, these studies have only focused on infinite-time stability, whereas in finite time, the performance of the systems cannot be guaranteed. Unlike conventional Lyapunov stability, the FTS can achieve superior transient performance in finite time. Wei et al. (2020) proposed a novel MDADT switching signal. The dynamic decomposition technique was used to generate the switching signals, and sufficient conditions for FTS were detailed. For nonlinear switched systems with time delay, the Lyapunov-Razumikhin approach and Lyapunov-Krasovskii function method were used to investigate FTS problems (Wang et al., 2020). Furthermore, the tracking control is widely applied in flight vehicles (Liu et al., 2021). The finite-time tracking control problems in Wang et al. (2017) furthers research on finite-time robust tracking control of switched flight vehicles.

The tracking control problem for uncertain systems is investigated as follows (Liu et al., 2019; Chen et al., 2020; Lu et al., 2022): (1) constant parameter control, such as robust control, proportional integral derivative control, and optimal control, in which the worst case is considered for the bounded uncertainties and disturbances; (2) variable parameter control, such as adaptive and observer-based controls, in which the uncertainties and disturbances are compensated in real time; (3) learning-based control policy, such as reinforcement learning, which compensates uncertainties without prior knowledge and learns a control law through trial and error. In constant parameter control, the model uncertainties and external disturbances are assumed to be bounded with known boundaries, which result in performance degradation and conservative control laws. The variable parameter control method can be used to mitigate the problem of time-varying uncertainties with unknown boundaries. However, the model uncertainties are assumed to be linearly parameterized with predefined structure and unknown time-varying parameters. The learning-based control method can be used for addressing system uncertainties with unknown boundaries and unknown structures (Yuan et al., 2017). However, this method cannot ensure stability, and computational complexities increase. A novel model-reference adaptive law and a switching logic were developed for uncertain switched systems. Ban et al. (2018) designed an *H*_∞_ controller for polytopic uncertain switched systems. Introducing scalar parameters reduced the conservatism of the linear matrix inequality (LMI) conditions and simultaneously ensured robust *H*_∞_ performance of the system. The problems of nonfragile control for nonlinear switched systems considering actuator failures and parametric uncertainties were studied in Sakthivel et al. (2018). The Lyapunov-Krasovskii function method and ADT approach were used to design a nonfragile reliable sampled-data controller. These studies have focused on control in the ideal environment. However, in practice, because of the limitation of network bandwidth, a network delay and packet loss always exist, which cause inevitable asynchronous switching. Thus, the control switching lags behind state switching. This phenomenon results in performance degradation and instability. Li and Deng (2018) investigated the *p*th moment exponential input-to-state stability (ISS) of the switched systems with asynchronous switching. The indefinite differentiable Lyapunov function was combined with ADT to establish the ISS conditions of the switched systems with Lévy noise. The conclusion of these results (Zhang and Zhu, 2019) were generalized in Li and Deng (2018), and the ISS problems, stochastic-ISS, and integral-ISS for asynchronously switched systems with asynchronous switching were investigated. Fast ADT switching was introduced to mitigate the increase in the Lyapunov-Krasovskii function when active subsystems matches the controller. However, in most existing results on controller design for flight vehicles, although stability and robustness can be attained, achieving optimal control performance in real-time challenging.

With improvement in the calculating ability of computing devices, machine learning has been widely applied in many fields, including the control field (Cheng and Zhang, 2018; Guo et al., 2019; Gheisarnejad and Khooban, 2021). Xu et al. (2019) proposed a model-driven DDPG algorithm for robotic multi-peg-in-hole assembly to avoid the analysis of the contact model. A feedback strategy and a fuzzy reward function were proposed to improve data efficiency and learning efficiency. In Tailor and Izzo (2019), optimal trajectory for a quadcopter model in two dimensions was investigated. A near-optimal policy was proposed to construct trajectories that satisfy Pontryagin’s principle of optimality through supervised learning. With improved aircraft performance, the guidance and control system require rapidity, stability, and robustness. Therefore, deep learning and the exploration of reinforcement learning are an effective solution to this problem, which cannot be solved using conventional control. Cheng et al. (2019) and Gaudet et al. (2020) studied the fuel-optimal landing problems based on DRL. The optional control algorithms were designed considering the uncertainties of environment and system parameters by using deep neural networks and policy gradient methods to ensure the real-time performance and optimality of the landing mission. The design of the reward function is a critical factor for controller/filter design with DRL. In this method, the final performance of the training networks was determined but not treated satisfactorily. This study is motivated to solve this problem.

However, the methods proposed in Tailor and Izzo (2019) and Gaudet et al. (2020) could not ensure the robustness and stability of the given system. Considering the advantages and limitations of the model-based and model-free methods, we proposed a novel nonfragile DRL for achieving asynchronously finite-time robust tracking control of switched flight vehicles. In this method, the best compromise was realized between system stability, robustness, and rapidity. The intelligent controller based on nonfragile *H*_∞_ control and DRL was proposed to compensate model uncertainties and realize superior control performance. The FTS and finite-time robustness were realized by nonfragile *H*_∞_ control, and the transient performance was optimized by using the adaptive deep deterministic policy gradient (ADDPG) algorithm. Because of the significance of reward function design in the training process, adaptive hyperparameters were introduced to construct a generalized reward function to improve the performance and achieve robustness. Therefore, the contributions of the paper can be summarized as follows:

1. A novel control structure consisting of dynamics-based and learning-based controllers was proposed for the finite-time tracking control of switched flight vehicles. The robust control is focused on the worst case of uncertainties. However, transient performance is not ensured. The learning-based method, such as DRL, can address uncertainties with unknown boundaries and structures. However, stability is not guaranteed. Compared with the conventional method, in such a design structure, the advantages of both conventional robust control method and pure DRL are combined. The DRL is used to enhance control performance without exploiting their structures or boundaries, and the robustness is guaranteed by using model-based robust control. Thus, an optimal compromise between robustness and dynamic performance was achieved.
2. The stability and robustness of closed-loop system were guaranteed by using non-fragile control theory. The restricted DRL algorithm was proposed, in which the boundaries of scheduling intervals were predefined. The scheduling of parameters can be viewed as the perturbation of parameters within a given interval. Compared with pure DRL, the proposed method improved training efficiency and ensured stability of the closed-loop system.
3. The adaptive reward functions were proposed to realize rapid training convergence. The reward functions were crucial for the DRL algorithm. The conventional method of reward functions typically depends on the designing experience of the researchers, which degrade training efficiency and result in trial and error. Therefore, in the proposed method, adaptive factors for reward functions were used to improve training efficiency.

The rest of the paper is organized as follows. In Section 2, the structure of intelligent switched controllers is presented. In Section 3, the finite-time robust tracking control algorithm using DRL and *H*_∞_ control was proposed. A numerical example is provided in Section 4. Finally, Section 5 presents the summary and directions for future studies.

## 2 Problem statement

The HiMAT vehicle was studied, which is an unmanned flight vehicle. Its nonlinear model can be described in Eq. (1).


(1) $$\left\{ \begin{array}{l} m_{f}\dot{v}=T\cos\alpha -D-m_{f}g\sin\left(\theta -\alpha \right)\\ \dot{\alpha }=\frac{-T\sin\alpha }{m_{f}v}-\frac{L}{m_{f}v}+q+\frac{g\cos\left(\theta -\alpha \right)}{v}\\ \dot{\varphi }=q\\ I_{y}\dot{q}=M_{yy}\\ \dot{h}=v\sin\left(\theta -\alpha \right)\\ \theta =\varphi -\alpha \end{array}\right.$$

where *m*_f_ and *v* denote the mass and velocity of the flight vehicle, respectively. Here, α , θ , φ , and *q* are the attack angle, flight path angle, pitch angle, and pitch rate, respectively. Furthermore, *M*_yy_ and *I*_y_ are the pitch moment and the moment of inertia about the pitch axis, respectively. Furthermore, *g* denotes the gravitational constant. The notations of T , D , and L represent the thrust, drag force, and lift force, which can be expressed as follows:


(2) $$\left\{ \begin{array}{l} T=QSC_{T}\\ D=QSC_{D}\\ L=QSC_{L} \end{array}\right.$$

where $C_{L}\left(\alpha \right)=C_{L0}^{0}+C_{L1}^{\alpha }\alpha$ , $C_{D}\left(\alpha \right)=C_{D1}^{0}+C_{D2}^{\alpha }\alpha +C_{D3}^{\alpha^{2}}\alpha^{2}$ , $C_{T}\left(\delta_{c}\right)=C_{T}^{0}+C_{T}^{\delta_{c}}\delta_{c}$ , $Q=0.5\rho V^{2}$ , in which ρ and $\delta_{c}$ are the air density and throttle setting.

Based on Jacobian linearization, the nonlinear model of HiMAT vehicle can be converted into the linear model to bridge the connection between complex nonlinear and linear models. Therefore, the longitudinal short-period model of the HiMAT vehicle can be modeled as switched systems as follows:


(3) $$\left\{ \begin{array}{l} x\left(k+1\right)=A_{i}x\left(k\right)+B_{i}u\left(k\right)+D_{i}\omega \left(k\right)\\ y\left(k\right)=C_{i}x\left(k\right) \end{array}\right.$$

where $x\left(k\right)={\left[\alpha \;q\right]}^{T}\in R^{x}$ is the state vector, $\omega \left(k\right)\in R^{\omega }$ represents the external disturbance that belongs to $L_{2}\left[0,\infty \right)$ , $u\left(k\right)={\left[\begin{array}{ccc} \delta_{e} & \delta_{v} & \delta_{c} \end{array}\right]}^{T}\in R^{u}$ with $\delta_{e}$ , $\delta_{v}$ , and $\delta_{c}$ representing the elevator, elevon, and canard deflection, and $y\left(k\right)\in R^{y}$ denoting the control and output signals. Here, $\sigma \left(k\right)=i\to \Omega =\left\{1,2,\cdots ,n\right\}$ is the switching function, which is a piecewise continuous constant function. Furthermore, n>1 is the number of subsystems. The characteristic of subsystems is assumed to depend on the switching signal, which are known previously. Here, $A_{i}$ , $B_{i}$ , $C_{i}$ , and $D_{i}$ are system matrices with appropriate dimensions.

In the network environment, because of the limit source of network bandwidth, the packet dropouts should be considered. The packet dropouts are considered in the channel of sensors–controllers to satisfy the Bernoulli distribution (Cheng et al., 2018). Therefore, the measured output is described as follows:


(4) $$\left\{ \begin{array}{l} \tilde{y}\left(k\right)=\theta \left(k\right)y\left(k\right)\\ \mathrm{Pr}ob\left\{\theta \left(k\right)=1\right\}=E\left\{\theta \left(k\right)\right\}=\rho \\ \mathrm{Pr}ob\left\{\theta \left(k\right)=0\right\}=1-E\left\{\theta \left(k\right)\right\}=1-\rho \end{array}\right.$$

where $\tilde{y}\left(k\right)$ is the measured output, $\theta \left(k\right)$ represents a stochastic variable satisfying the Bernoulli distribution and takes value of $\left\{0,1\right\}$ , and $\rho \in \left[0,1\right]$ is the probability of packet dropouts.

**Figure 1:**
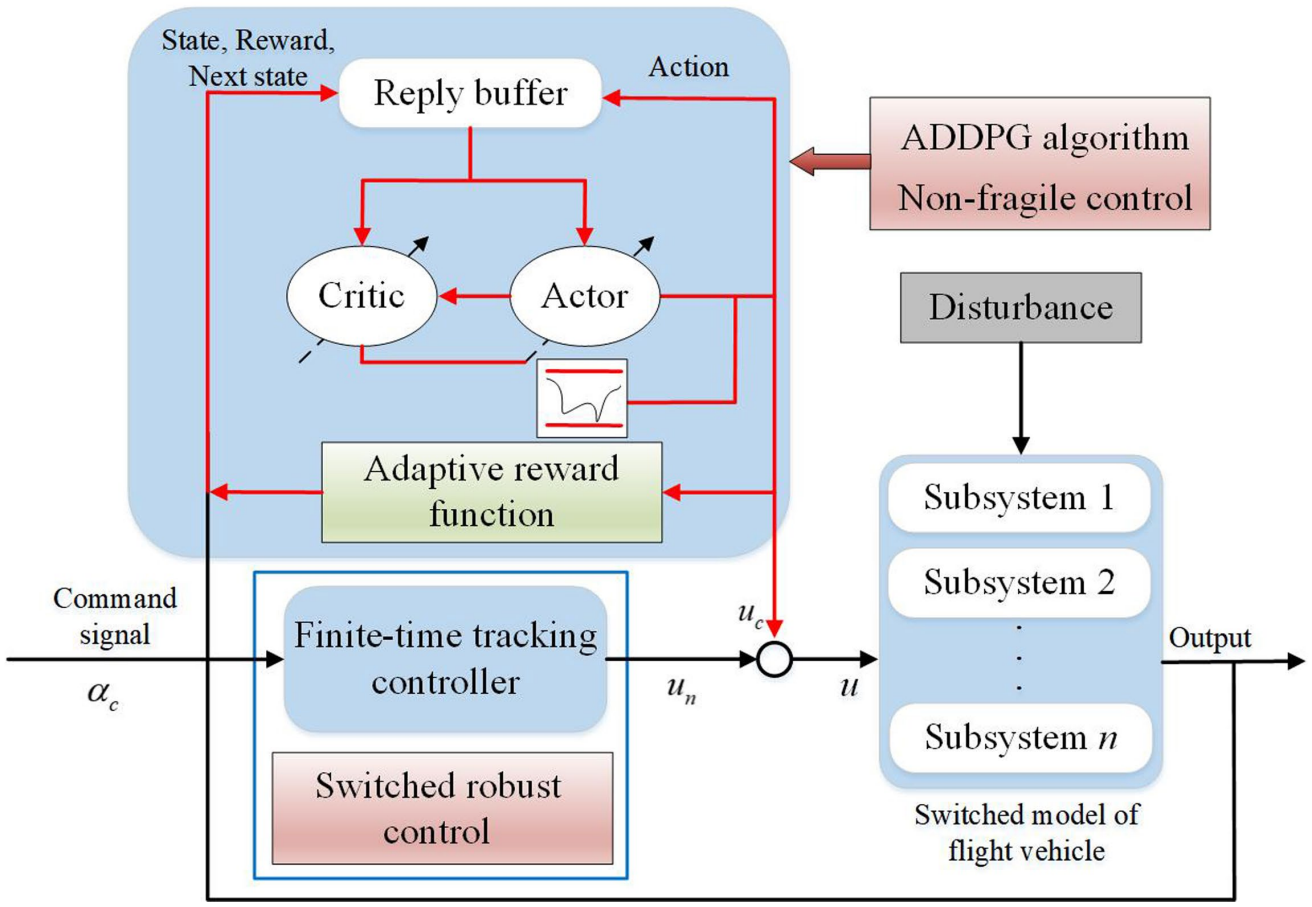
Structure of the controller.

The control structure of switched flight vehicles to ensure stability and improve transient performance is displayed in Figure 1.

The controller diagram reveals that the controller is composed of two parts:


(5) $$u\left(k\right)=u_{n}\left(k\right)+u_{c}\left(k\right)$$

where $u_{n}\left(k\right)$ is the dynamics-based controller, and ***u***_c_ is the learning-based controller, which are developed based on finite-time $H_{\infty }$ control and DRL. The FTS and prescribed attenuation index are ensured by $u_{n}\left(k\right)$ , whose parameters can be obtained by the LMI technique. The transient performance is improved by $u_{c}\left(k\right)$ , whose parameters are scheduled by the ADDPG algorithm.

The tracking error of the output is defined as $e\left(k\right)=r_{c}\left(k\right)-y\left(k\right)$ , and the objective of tracking control is as follows:


(6) $$\lim_{k\to \infty }e\left(k\right)=0$$

where $r_{c}\left(k\right)$ denotes the command signal.

We set the integral of tracking error as follows:


(7) $$g\left(k\right)=\overset{k-1}{\underset{l=0}{\sum }}e\left(l\right)=\overset{k-1}{\underset{l=0}{\sum }}\left(r_{c}\left(l\right)-y\left(l\right)\right)$$

The feedback controller is proposed as follows:


(8) $$u_{n}\left(k\right)=K_{n,i}\tilde{x}\left(k\right)=K_{n,1i}x\left(k\right)+K_{n,2i}g\left(k\right)$$

where $\tilde{x}\left(k\right)={\left[\begin{array}{cc} x{\left(k\right)}^{T} & g{\left(k\right)}^{T} \end{array}\right]}^{T}$ , $K_{n,1i}$ and $K_{n,2i}$ are the gain matrices to be determined.

Nominal controller parameters $K_{n,1i}$ and $K_{n,2i}$ can be designed by the $H_{\infty }$ control, the variation internal of learning-based controller $u_{c}\left(k\right)$ in subsystem i can be perceived as the additional bounded uncertainties of the dynamics-based controller. Thus, the parameters vary in the interval $\left[K_{n,i}-\Delta {\underline{K}}_{c,i},K_{n,i}+\Delta {\bar{K}}_{c,i}\right]$ and the stability of learning-based controller can be analyzed by using nonfragile control theory. Here, $\Delta K_{c,i}$ is defined as the additional compensation to obtain the actual gain matrices as follows:


(9) $$K_{i}=K_{n,i}+\Delta K_{c,i}$$

where $\Delta {\underline{K}}_{c,i}$ and $\Delta {\bar{K}}_{c,i}$ denote the lower and upper bounds of $\Delta K_{c,i}$ ; set $\Delta K_{c,i}=M_{i}F_{i}N_{i}$ , $M_{i}$ and $N_{i}$ are known parameters with appropriate dimensions, and $F_{i}$ are uncertain matrices satisfying the following equation:


(10) $$F_{i}^{T}F_{i}\leq I$$

*Remark 1*: The model of flight vehicle can be given based on switched systems. The variation of states in the envelope can be viewed as the switching between subsystems. The tracking controller is composed of two parts, namely dynamics-based controller $u_{n}\left(k\right)$ , which is developed based on finite-time $H_{\infty }$ control to ensure stability and prescribed attenuation index; the learning-based controller $u_{c}\left(k\right)$ , which is based on ADDPG algorithm to achieve superior performance in real time. The output of $u_{c}\left(k\right)$ varies in the neighbor interval of $u_{n}\left(k\right)$ with given bounds. Therefore, the nonfragile control can be used to ensure the stability of $u_{c}\left(k\right)$ . As mentioned, ensuring stability, robustness, and optimal performance simultaneously remains difficult. To improve training efficiency, adaptive factors for reward functions were applied in DDPG algorithm. With inspiration from the achievements in the DDPG algorithm and robust control, the advantages of model-based method ( $H_{\infty }$ control) and model-free method (DRL) were considered the problem.

*Remark 2*: The compensation of learning-based controller is considered as an additional gain value on the controller parameters with known bounds, which can be predefined and can presented by $M_{i}$ and $N_{i}$ . The optimal control policy can be realized in the scheduling interval by using the ADDPG algorithm.

The switching of controller always lags the switching of system mode because of packet dropouts. The i th subsystem is assumed to be activated at $k_{i}$ , and the controller of i th subsystem is activated at $k_{i}+\Delta_{i}$ , where $\Delta_{i}$ denotes the length of unmatched periods. The condition in which unmatched and matched periods exist simultaneously is called asynchronous switching. The Lyapunov-like function decreases in matched periods and increases in unmatched periods with bounded rates, where $a_{i}$ are introduced to represent the decreasing rate in matched periods, and $b_{i}$ represent the increasing rate in unmatched periods. The increasing coefficients of the Lyapunov-like function at switching instants are set to be $\mu_{i}$ .

For proof, the following assumptions are introduced.

*Assumption 1* (Cheng et al., 2017): For given positive constant $N_{f}$ , the time-varying exogenous disturbance $\omega \left(k\right)$ satisfies the following equation:


(11) $$\overset{N_{f}}{\underset{k=0}{\sum }}\omega^{T}\left(k\right)\omega \left(k\right)\leq \bar{\omega }$$

where $\bar{\omega }$ is the upper bound of external disturbance.

*Assumption 2* (Cheng et al., 2017): The maximum number of consecutive data missing is set to be *N*_1_, and the maximum probability of data missing is set to be $\bar{\rho }$ .

According to the aforementioned statement, the closed-loop switched systems can be described as follows:


(12) $$\begin{array}{l} \left\{\begin{array}{l} \tilde{x}\left(k+1\right)={\tilde{A}}_{ii}\tilde{x}\left(k\right)+\tilde{\theta }\left(k\right){\tilde{A}}_{1i}\tilde{x}\left(k\right)+{\tilde{B}}_{i}\tilde{\omega }\left(k\right)\\ e\left(k\right)={\tilde{C}}_{i}\tilde{x}\left(k\right)+\tilde{\theta }\left(k\right){\tilde{C}}_{1i}\tilde{x}\left(k\right)+{\tilde{D}}_{i}\tilde{\omega }\left(k\right) \end{array},\forall k\in [k_{i}+\Delta_{i},k_{i+1}\right)\\ \left\{\begin{array}{l} \tilde{x}\left(k+1\right)={\tilde{A}}_{ij}\tilde{x}\left(k\right)+\tilde{\theta }\left(k\right){\tilde{A}}_{1i}\tilde{x}\left(k\right)+{\tilde{B}}_{i}\tilde{\omega }\left(k\right)\\ e\left(k\right)={\tilde{C}}_{i}\tilde{x}\left(k\right)+\tilde{\theta }\left(k\right){\tilde{C}}_{1i}\tilde{x}\left(k\right)+{\tilde{D}}_{i}\tilde{\omega }\left(k\right) \end{array},\forall k\in [k_{i},k_{i}+\Delta_{i}\right) \end{array}$$

where $\tilde{\omega }\left(k\right)={\left[\begin{array}{cc} \omega^{T}\left(k\right) & r_{c}^{T}\left(k\right) \end{array}\right]}^{T}\text{,}$ ${\tilde{A}}_{ii}=\left[\begin{array}{cc} A_{i}+B_{i}K_{1i} & B_{i}K_{2i}\\ -\rho C_{i} & I \end{array}\right]\text{,}$ ${\tilde{A}}_{1i}=\left[\begin{array}{cc} 0 & 0\\ -C_{i} & 0 \end{array}\right]\text{,}$ ${\tilde{A}}_{ij}=\left[\begin{array}{cc} A_{i}+B_{i}K_{1j} & B_{i}K_{2j}\\ -\rho C_{i} & I \end{array}\right]\text{,}$ ${\tilde{B}}_{i}=\left[\begin{array}{cc} D_{i} & 0\\ 0 & I \end{array}\right]$ ${\tilde{C}}_{i}=\left[\begin{array}{cc} -\rho C_{i} & 0 \end{array}\right]\text{,}$ ${\tilde{C}}_{1i}=\left[\begin{array}{cc} -C_{i} & 0 \end{array}\right]\text{,}$ ${\tilde{D}}_{i}=\left[\begin{array}{cc} 0 & I \end{array}\right]\text{,}$ $\tilde{\theta }\left(k\right)=\theta \left(k\right)-\rho$ .

Furthermore, the definitions of finite-time stable, finite-time boundedness, and finite-time *H*_∞_ performance for switched systems are expressed as follows:

*Definition 1* (Wei et al., 2020): For given appropriate constant positive matrix $R_{s}$ , positive constants $c_{1}>0$ , $c_{2}>0$ , and $N_{f}$ with $c_{1}<c_{2}$ , respectively. The switched systems in Eq. (12) with $u\left(k\right)\text{≡}0$ and $\omega \left(k\right)\text{≡}0$ are finite-time stable with respect to $\left(c_{1},c_{2},N_{f},R_{s}\right)$ if Eq. (13) holds.


(13) $$x^{T}\left(k_{0}\right)R_{s}x\left(k_{0}\right)\leq c_{1}\Rightarrow x^{T}\left(k\right)R_{s}x\left(k\right)\leq c_{2},\forall k\in \left\{1,2,\ldots ,N_{f}\right\}$$

*Definition 2* (Wei et al., 2020): For given appropriate constant positive matrix $R_{s}$ , constants $c_{1}>0$ , $c_{2}>0$ , $\bar{\omega }$ , and $N_{f}$ with $c_{1}<c_{2}$ , respectively. The switched system in Eq. (12) is finite-time bounded (FTB) with respect to $\left(c_{1},c_{2},\bar{\omega },N_{f},R_{s}\right)$ such that the following expression holds:


(14) $$x^{T}\left(k_{0}\right)R_{s}x\left(k_{0}\right)\leq c_{1}\Rightarrow x^{T}\left(k\right)R_{s}x\left(k\right)\leq c_{2},\forall k\in \left\{1,2,\ldots ,N_{f}\right\}$$

where the external disturbance satisfies Assumption 1.

*Definition 3* (Wei et al., 2020): For a given appropriate constant positive matrix $R_{s}$ , constants $c_{1}>0$ , $c_{2}>0$ for $\bar{\omega }$ and $N_{f}$ with $c_{1}<c_{2}$ . The system in Eq. (12) exhibits finite-time $H_{\infty }$ performance $\gamma_{d}$ if the system is FTB and satisfies the following expression:


(15) $$\overset{N_{f}}{\underset{s=0}{\sum }}e^{T}\left(s\right)e\left(s\right)\leq \gamma_{d}^{2}\overset{N_{f}}{\underset{s=0}{\sum }}{\tilde{\omega }}^{T}\left(s\right)\tilde{\omega }\left(s\right)$$

Thus, the main purposes of controller design is to ensure that the switched system is FTS with prescribed $H_{\infty }$ performance $\gamma_{d}$ with respect to $\left(c_{1},c_{2},\bar{\omega },N_{f},R_{s}\right)$ , which is equivalent to design the robust controller, such that the following condition is satisfied:

1. The switched systems in Eq. (12) is FTB.
2. For given constant $\gamma_{d}>0$ , the system in Eq. (12) satisfies Eq. (15) under zero-initial situation for all external disturbance satisfies Eq. (11).

Based on the structure of control diagram, the design process is categorized into two steps:

*Step 1*: The scheduling interval of control parameters can be assumed to be the uncertain compensation of dynamics-based controller. Considering the controller uncertainties and asynchronous switching caused by packet dropouts, the finite-time $H_{\infty }$ controllers are derived as dynamics-based controller according to nonfragile control theory and finite-time robust control theory in terms of LMI.

*Step 2*: The variations of controller parameters are assumed to be the action, and the dynamic model of flight vehicles is assumed to be the environment. The DRL algorithm was introduced to derive the learning-based controller to realize optimal control policy, in which the ADDPG algorithm was proposed as the model-free method in the actor–critic framework.

## 3 Main results

A dynamics-based controller was proposed to ensure stability and a prescribed performance index. The ADDPG algorithm was developed to realize performance and ensure controllers can adaptively schedule parameters.

### 3.1 Dynamics-based controller design

*Definition 4* (Zhao et al., 2017): Given switching signal $\sigma \left(k\right)$ and any $0\leq k_{1}\leq k_{2}$ , let $N_{\sigma i}\left(k_{1},k_{2}\right)$ be the activated number of i th subsystem over the time interval $\left(k_{1},k_{2}\right)$ . Here, $T_{i}\left(k_{1},k_{2}\right)$ denotes the total running time of i th subsystem during the time interval $\left(k_{1},k_{2}\right)$ , i∈Ω . If positive numbers $N_{0i}$ and $\tau_{ai}$ , exist such that


(16) $$N_{\sigma i}\left(k_{1},k_{2}\right)\leq N_{0i}+\frac{T_{i}\left(k_{1},k_{2}\right)}{\tau_{ai}}$$

then $\tau_{ai}$ is called the MDADT and $N_{0i}$ is called the mode-dependent chatter bounds.

*Lemma 1* (Cheng et al., 2017): For given symmetric matric Y , matrices F , $\tilde{M}$ , and $\tilde{N}$ , if a scalar ε>0 exists such that


(17) $$Y+\varepsilon^{-1}{\tilde{M}}^{T}\tilde{M}+\varepsilon {\tilde{N}}^{T}\tilde{N}<0$$

then we can obtain the following:


(18) $$Y+{\tilde{M}}^{T}F\tilde{N}+{\tilde{N}}^{T}F^{T}\tilde{M}<0$$

where F satisfies $F^{T}F<I$ .

*Lemma 2* (Aristidou et al., 2014): For given matrix ***Q***, which satisfies


(19) $$Q=\left[\begin{array}{cc} Q_{11} & Q_{12}\\ Q_{21} & Q_{22} \end{array}\right]$$

where $Q_{12}=Q_{21}^{T}$ , and ***Q***_11_ and ***Q***_22_ are invertible matrices. Then we can conclude that the following three conditions are equivalent, which is called Schur Complement.


$$\begin{array}{l} \left(1\right)\;Q<0;\\ \left(2\right)\;Q_{11}<0,Q_{22}-Q_{12}^{T}Q_{11}^{T}Q_{12}<0;\\ \left(3\right)\;Q_{22}<0,Q_{11}-Q_{12}Q_{11}^{T}Q_{12}^{T}<0. \end{array}$$

*Theorem 1*: Given system Eq. (12) and constant scalars $0<a_{i}<1$ , $b_{i}>0$ , $\mu_{i}\geq 1$ , γ>0 , if matrices $S_{i}>0$ , $S_{j}>0$ , $S_{ij}>0$ , and $W_{i}$ , ∀i,j∈Ω,i≠j , then the following expression is obtained:


(20) $$S_{j}\leq \mu_{i}S_{i}$$

(21) $$\left[\begin{array}{cccc} -S_{i} & 0 & {\tilde{A}}_{ii}S_{i} & {\tilde{B}}_{i}\\ {}^{*} & -S_{i} & \tilde{\rho }{\tilde{A}}_{1i}S_{i} & 0\\ {}^{*} & {}^{*} & -\left(1-a_{i}\right)S_{i} & 0\\ {}^{*} & {}^{*} & {}^{*} & -\gamma^{2}W_{i} \end{array}\right]<0$$

(22) $$\left[\begin{array}{cccc} -S_{ij} & 0 & {\tilde{A}}_{ij}S_{j} & {\tilde{B}}_{i}\\ {}^{*} & -S_{ij} & \tilde{\rho }{\tilde{A}}_{1i}S_{j} & 0\\ {}^{*} & {}^{*} & -\left(1+b_{i}\right)\left(S_{ij}-S_{j}-S_{j}^{T}\right) & 0\\ {}^{*} & {}^{*} & {}^{*} & -\gamma^{2}W_{i} \end{array}\right]<0$$

then the switched system in Eq. (12) is FTB with respect to $\left(c_{1},c_{2},\bar{\omega },N_{f},R_{s}\right)$ if the MDADT satisfies the following equations:


(23) $$\tau_{\mathrm{ai}}\geq \tau_{\mathrm{ai}}^{*}=\frac{N_{f}\ln\mu_{i}+N_{f}\Delta_{i}\ln\hbar_{i}}{\ln\frac{c_{2}}{\eta_{1}}-\ln\left[\frac{c_{1}}{\eta_{2}}+\gamma^{2}{\tilde{b}}_{\text{max}}^{N_{f}}\eta_{3}\bar{\omega }\right]-N_{f}\ln{\tilde{a}}_{i}}$$

(24) $$\left(\frac{c_{1}}{\eta_{2}}+\gamma^{2}{\tilde{b}}_{\text{max}}^{N_{f}}\eta_{3}\bar{\omega }\right){{\tilde{a}}_{i}}^{N_{f}}\leq \frac{c_{2}}{\eta_{1}}$$

where $\eta_{1}=\max_{i\in \Omega }\left(\lambda_{\text{max}}\left({\bar{S}}_{i}\right),\lambda_{\text{max}}\left({\bar{S}}_{ij}\right)\right)\text{,}$ $\eta_{2}=\min_{i\in \Omega }\left(\lambda_{\text{min}}\left({\bar{S}}_{i}\right),\lambda_{\text{min}}\left({\bar{S}}_{ij}\right)\right)\text{,}$ $\eta_{3}=\lambda_{\text{max}}\left(W_{i}\right)\text{,}$ $\tilde{\rho }=\sqrt{\rho \left(1-\rho \right)}\text{,}$ ${\bar{S}}_{i}=R_{s}^{1/2}S_{i}R_{s}^{1/2}\text{,}$ ${\bar{S}}_{j}=R_{s}^{1/2}S_{j}R_{s}^{1/2}\text{,}$ ${\tilde{a}}_{i}=1-a_{i}\text{,}$ ${\tilde{b}}_{i}=1+b_{i}\text{,}$ $\hbar_{i}={\tilde{b}}_{i}/{\tilde{a}}_{i}\text{,}$ ${\tilde{b}}_{\text{max}}=\max\left\{{\tilde{b}}_{i}\right\}$ .

Proof: For positive constant k , we define $k_{0}=0$ and $k_{1},k_{2},\ldots ,k_{i},k_{i+1},\ldots k_{n}$ as the switching instants over the interval $\left[0,k\right]$ , suppose the following Lyapunov functions exist:


(25) $$V_{i}\left(k\right)={\tilde{x}}^{T}\left(k\right)P_{i}\tilde{x}\left(k\right)$$

Class $\kappa_{\infty }$ functions exist as follows:


(26) $$\kappa_{1i}\left|\tilde{x}\left(k\right)\right|\leq V_{i}\left(k\right)\leq \kappa_{2i}\left|\tilde{x}\left(k\right)\right|$$

(27) $$\Delta V_{i}\left(k\right)\leq \{\begin{array}{l} -a_{i}V_{i}\left(k\right),\forall k\in \left[k_{0},k_{1}\right]\cup \left[k_{i}+\Delta_{i},k_{i+1}\right]\\ b_{i}V_{i}\left(k\right),\forall k\in \left[k_{i},k_{i}+\Delta_{i}\right] \end{array}$$

(28) $$V_{i}\left(k\right)\leq \mu_{i}V_{j}\left(k\right)$$

where $P_{i}>0$ are Lyapunov matrices.

Define $\xi \left(k\right)={\left[\begin{array}{cc} {\tilde{x}}^{T}\left(k\right) & {\tilde{\omega }}^{T}\left(k\right) \end{array}\right]}^{T}\text{,}$ and combining with Eqs. (12) and (27), we can obtain the following expression:


(29) $$\begin{array}{l} \;\Delta V_{i}\left(k\right)+a_{i}V_{i}\left(k\right)-\gamma^{2}{\tilde{\omega }}^{T}\left(k\right)W_{i}\tilde{\omega }\left(k\right)\\ =V_{i}\left(k+1\right)-V_{i}\left(k\right)+a_{i}V_{i}\left(k\right)-\gamma^{2}{\tilde{\omega }}^{T}\left(k\right)W_{i}\tilde{\omega }\left(k\right)\\ =E\left\{{\tilde{x}}^{T}\left(k+1\right)P_{i}\tilde{x}\left(k+1\right)\right\}-\left(1-a_{i}\right){\tilde{x}}^{T}\left(k\right)P_{i}\tilde{x}\left(k\right)\\ \;-\gamma^{2}{\tilde{\omega }}^{T}\left(k\right)W_{i}\tilde{\omega }\left(k\right)\\ =\xi^{T}\left(k\right)\left\{\begin{array}{l} \left[\begin{array}{l} {\tilde{A}}_{ii}^{T}\\ {\tilde{B}}_{i}^{T} \end{array}\right]P_{i}\left[\begin{array}{cc} {\tilde{A}}_{ii} & {\tilde{B}}_{i} \end{array}\right]+{\tilde{\rho }}^{2}\left[\begin{array}{c} {\tilde{A}}_{1i}^{T}\\ 0 \end{array}\right]P_{i}\left[\begin{array}{cc} {\tilde{A}}_{1i} & 0 \end{array}\right]\\ +\left[\begin{array}{cc} -\left(1-a_{i}\right)P_{i} & 0\\ 0 & -\gamma^{2}W_{i} \end{array}\right] \end{array}\right\}\xi \left(k\right)\\ =\xi^{T}\left(k\right)\Pi_{ii}\xi \left(k\right) \end{array}$$

(30) $$\begin{array}{l} \;\Delta V_{i}\left(k\right)-b_{i}V_{i}\left(k\right)-\gamma^{2}{\tilde{\omega }}^{T}\left(k\right)W\tilde{\omega }\left(k\right)\\ =V_{i}\left(k+1\right)-V_{i}\left(k\right)-b_{i}V_{i}\left(k\right)-\gamma^{2}{\tilde{\omega }}^{T}\left(k\right)W_{i}\tilde{\omega }\left(k\right)\\ =E\left\{{\tilde{x}}^{T}\left(k+1\right)P_{ij}\tilde{x}\left(k+1\right)\right\}-\left(1+b_{i}\right)x^{T}\left(k\right)P_{ij}x\left(k\right)\\ \;-\gamma^{2}{\tilde{\omega }}^{T}\left(k\right)W\tilde{\omega }\left(k\right)\\ =\xi^{T}\left(k\right)\left\{\begin{array}{l} \left[\begin{array}{l} {\tilde{A}}_{ij}^{T}\\ {\tilde{B}}_{i}^{T} \end{array}\right]P_{j}\left[\begin{array}{cc} {\tilde{A}}_{ij} & {\tilde{B}}_{i} \end{array}\right]+{\tilde{\rho }}^{2}\left[\begin{array}{c} {\tilde{A}}_{1i}^{T}\\ 0 \end{array}\right]P_{i}\left[\begin{array}{cc} {\tilde{A}}_{1i} & 0 \end{array}\right]\\ +\left[\begin{array}{cc} -\left(1+b_{i}\right)P_{ij} & 0\\ 0 & -\gamma^{2}W_{i} \end{array}\right] \end{array}\right\}\xi \left(k\right)\\ =\xi^{T}\left(k\right)\Pi_{ij}\xi \left(k\right) \end{array}$$

where


$$\Pi_{ii}=\left[\begin{array}{cccc} -P_{i} & 0 & P_{i}{\tilde{A}}_{ii} & P_{i}{\tilde{B}}_{i}\\ {}^{*} & -P_{i} & \tilde{\rho }P_{i}{\tilde{A}}_{1i} & 0\\ {}^{*} & {}^{*} & -\left(1-a_{i}\right)P_{i} & 0\\ {}^{*} & {}^{*} & {}^{*} & -\gamma^{2}W_{i} \end{array}\right]\text{,}$$

$$\Pi_{ij}=\left[\begin{array}{cccc} -P_{ij} & 0 & P_{ij}{\tilde{A}}_{ij} & P_{ij}{\tilde{B}}_{i}\\ {}^{*} & -P_{ij} & \tilde{\rho }P_{ij}{\tilde{A}}_{1i} & 0\\ {}^{*} & {}^{*} & -\left(1+b_{i}\right)P_{ij} & 0\\ {}^{*} & {}^{*} & {}^{*} & -\gamma^{2}W_{i} \end{array}\right]\text{.}$$

Setting $S_{i}=P_{i}^{-1}$ and performing a congruence transformation to Eqs. (29), (30) by matrices $\mathrm{diag}\left\{S_{i},S_{i},S_{i},I\right\}$ and $\mathrm{diag}\left\{S_{ij},S_{ij},S_{j},I\right\}$ , we can obtain the following expression:


(31) $$\left[\begin{array}{cccc} -S_{i} & 0 & {\tilde{A}}_{ii}S_{i} & {\tilde{B}}_{i}\\ {}^{*} & -S_{i} & \tilde{\rho }{\tilde{A}}_{1i}S_{i} & 0\\ {}^{*} & {}^{*} & -\left(1-a_{i}\right)S_{i} & 0\\ {}^{*} & {}^{*} & {}^{*} & -\gamma^{2}W_{i} \end{array}\right]<0$$

(32) $$\left[\begin{array}{cccc} -S_{ij} & 0 & {\tilde{A}}_{ij}S_{j} & {\tilde{B}}_{i}\\ {}^{*} & -S_{ij} & \tilde{\rho }{\tilde{A}}_{1i}S_{j} & 0\\ {}^{*} & {}^{*} & -\left(1+b_{i}\right)S_{j}^{T}S_{ij}^{-1}S_{j} & 0\\ {}^{*} & {}^{*} & {}^{*} & -\gamma^{2}W_{i} \end{array}\right]<0$$

The inequality ${\left(S_{ij}-S_{j}\right)}^{T}S_{ij}\left(S_{ij}-S_{j}\right)\geq 0$ implies the following:


(33) $$S_{ij}-S_{j}-S_{j}^{T}\geq -S_{j}^{T}S_{ij}^{-1}S_{j}$$

We can conclude that Eq. (31) is equivalent to Eq. (21) and Eq. (32) is equivalent to Eq. (22), such that the following expression holds true:


(34) $$\Delta V_{i}\left(k\right)\leq \{\begin{array}{l} -a_{i}V_{i}\left(k\right)+\gamma^{2}{\tilde{\omega }}^{T}\left(k\right)\tilde{\omega }\left(k\right),\forall k\in \left[k_{0},k_{1}\right]\cup \left[k_{i}+\Delta_{i},k_{i+1}\right]\\ b_{i}V_{i}\left(k\right)+\gamma^{2}{\tilde{\omega }}^{T}\left(k\right)\tilde{\omega }\left(k\right),\forall k\in \left[k_{i},k_{i}+\Delta_{i}\right] \end{array}$$

Combining Eqs. (25), (26), (28), (34), we can obtain the following equations by iteration operation:

With the definitions of $\eta_{1}$ and $\eta_{2}$ , we have the following expression:


(35) $$\begin{array}{c} V_{\sigma \left(k\right)}\left(k\right)\leq {\tilde{a}}_{\sigma \left(k_{i}+\Delta_{i}\right)}^{k-k_{i}-\Delta_{i}}V_{\sigma \left(k_{i}+\Delta_{i}\right)}\left(k_{i}+\Delta_{i}\right)+\gamma^{2}\sum_{s=k_{i}+\Delta_{i}}^{k-1}{\tilde{a}}_{\sigma \left(k_{i}\right)}^{k-1-s}{\tilde{\omega }}^{T}\left(s\right)W_{i}\tilde{\omega }\left(s\right)\\ \leq \mu_{\sigma \left(k_{i}\right)}{\tilde{a}}_{\sigma \left(k_{i}+\Delta_{i}\right)}^{k-k_{i}-\Delta_{i}}{\tilde{b}}_{\sigma \left(k_{i}\right)}^{\Delta_{i}}V_{\sigma \left(k_{i}\right)}{\left(k_{i}+\Delta_{i}\right)}_{-}+\gamma^{2}\sum_{s=k_{i}+\Delta_{i}}^{k-1}{\tilde{a}}_{\sigma \left(k_{i}\right)}^{k-1-s}{\tilde{\omega }}^{T}\left(s\right)W_{i}\tilde{\omega }\left(s\right)+\gamma^{2}\sum_{s=k_{i}}^{k_{i}+\Delta_{i}-1}{\tilde{a}}_{\sigma \left(k_{i}\right)}^{k-k_{i}-\Delta_{i}}{\tilde{b}}_{\sigma \left(k_{i}\right)}^{k_{i}+\Delta_{i}-s-1}{\tilde{\omega }}^{T}\left(s\right)W_{i}\tilde{\omega }\left(s\right)\\ =\mu_{\sigma \left(k_{i}\right)}{\tilde{a}}_{\sigma \left(k_{i}+\Delta_{i}\right)}^{k-k_{i}}\hbar_{\sigma \left(k_{i}\right)}^{\Delta_{i}}V_{\sigma \left(k_{i}\right)}{\left(k_{i}+\Delta_{i}\right)}_{-}+\gamma^{2}\sum_{s=k_{i}+\Delta_{i}}^{k-1}{\tilde{a}}_{\sigma \left(k_{i}\right)}^{k-1-s}{\tilde{\omega }}^{T}\left(s\right)W_{i}\tilde{\omega }\left(s\right)+\gamma^{2}\sum_{s=k_{i}}^{k_{i}+\Delta_{i}-1}{\tilde{a}}_{\sigma \left(k_{i}\right)}^{k-s-1}\hbar_{\sigma \left(k_{i}\right)}^{k_{i}+\Delta_{i}-s-1}{\tilde{\omega }}^{T}\left(s\right)W_{i}\tilde{\omega }\left(s\right)\\ \leq \mu_{\sigma \left(k_{i}\right)}{\tilde{a}}_{\sigma \left(k_{i}\right)}^{k-k_{i}}\hbar_{\sigma \left(k_{i}\right)}^{\Delta_{i}}V_{\sigma \left(k_{i-1}\right)}\left(k_{i}\right)+\gamma^{2}\sum_{s=k_{i}}^{k-1}{\tilde{b}}_{\sigma \left(k_{i}\right)}^{k-1-s}{\tilde{\omega }}^{T}\left(s\right)W_{i}\tilde{\omega }\left(s\right)\\ \leq \mu_{\sigma \left(k_{i}\right)}{\tilde{a}}_{\sigma \left(k_{i}\right)}^{k-k_{i}}\hbar_{\sigma \left(k_{i}\right)}^{\Delta_{i}}\left[\mu_{\sigma \left(k_{i-1}\right)}{\tilde{a}}_{\sigma \left(k_{i-1}\right)}^{k_{i}-k_{i-1}}\hbar_{\sigma \left(k_{i-1}\right)}^{\Delta_{i-1}}V_{\sigma \left(k_{i-1}\right)}\left(k_{i-1}\right)+\gamma^{2}\sum_{s=k_{i-1}+\Delta_{i-1}}^{k_{i}-1}{\tilde{b}}_{\sigma \left(k_{i-1}\right)}^{k_{i}-1-s}{\tilde{\omega }}^{T}\left(s\right)W_{i}\tilde{\omega }\left(s\right)\right]+\gamma^{2}\sum_{s=k_{i}}^{k-1}{\tilde{b}}_{\sigma \left(k_{i}\right)}^{k-1-s}{\tilde{\omega }}^{T}\left(s\right)W_{i}\tilde{\omega }\left(s\right)\\ \leq \cdots \\ \leq {\tilde{a}}_{i}^{T_{i}\left(k_{0},k\right)}{\tilde{a}}_{i-1}^{T_{i-1}\left(k_{0},k\right)}\cdots {\tilde{a}}_{1}^{T_{1}\left(k_{0},k\right)}\hbar_{i}^{\Delta_{i}N_{\sigma ,i}\left(k_{0},k\right)}\hbar_{i-1}^{\Delta_{i-1}N_{\sigma ,i-1}\left(k_{0},k\right)}\cdots \hbar_{1}^{\Delta_{1}N_{\sigma ,1}\left(k_{0},k\right)}\mu_{i}^{N_{\sigma ,i}\left(k_{0},k\right)}\mu_{i-1}^{N_{\sigma ,i-1}\left(k_{0},k\right)}\cdots \mu_{1}^{N_{\sigma ,1}\left(k_{0},k\right)}V_{\sigma \left(k_{0}\right)}\left(k_{0}\right)\\ +{\tilde{a}}_{i}^{T_{i}\left(k_{0},k\right)}\cdots {\tilde{a}}_{1}^{T_{1}\left(k_{0},k\right)}\hbar_{i}^{\Delta_{i}N_{\sigma ,j}\left(k_{0},k\right)}\cdots \hbar_{1}^{\Delta_{1}N_{\sigma ,1}\left(k_{0},k\right)}\mu_{i}^{N_{\sigma ,i}\left(k_{0},k\right)}\mu_{i-1}^{N_{\sigma ,i-1}\left(k_{0},k\right)}\cdots \mu_{1}^{N_{\sigma ,1}\left(k_{0},k\right)}\gamma^{2}\sum_{s=k_{0}+\Delta_{0}}^{k_{1}-1}{\tilde{b}}_{\sigma \left(k_{0}\right)}^{k_{1}-1-s}{\tilde{\omega }}^{T}\left(s\right)W_{i}\tilde{\omega }\left(s\right)\\ +\cdots +\gamma^{2}\sum_{s=k_{i}+\Delta_{i}}^{k-1}{\tilde{b}}_{i}^{k-1-s}{\tilde{\omega }}^{T}\left(s\right)W_{i}\tilde{\omega }\left(s\right)\\ \leq \prod_{i=1}^{n}\mu_{i}^{N_{\sigma ,i}\left(k_{0},k\right)}{\tilde{a}}_{i}^{T_{i}\left(k_{0},k\right)}\hbar_{i}^{\Delta_{i}N_{\sigma ,i}\left(k_{0},k\right)}V_{\sigma \left(k_{0}\right)}\left(k_{0}\right)+\gamma^{2}{\tilde{b}}_{\text{max}}^{N_{f}}\sum_{s=k_{0}}^{k-1}\left(\prod_{i=1}^{n}\mu_{i}^{N_{\sigma ,i}\left(s,k\right)}{\tilde{a}}_{i}^{T_{i}\left(s,k\right)}\hbar_{i}^{\Delta_{i}N_{\sigma ,i}\left(s,k\right)}\right){\tilde{\omega }}^{T}\left(s\right)W_{i}\tilde{\omega \;}\left(s\right)\\ \leq \prod_{i=1}^{n}\mu_{i}^{N_{\sigma ,i}\left(k_{0},k\right)}{\tilde{a}}_{i}^{T_{i}\left(k_{0},k\right)}\hbar_{i}^{\Delta_{i}N_{\sigma ,i}\left(k_{0},k\right)}\left[V_{\sigma \left(k_{0}\right)}\left(k_{0}\right)+\gamma^{2}{\tilde{b}}_{\text{max}}^{N_{f}}\eta_{3}\bar{\omega }\right] \end{array}$$

(36) $$\begin{array}{c} V_{\sigma \left(k_{0}\right)}\left(k_{0}\right)={\tilde{x}}^{T}\left(k_{0}\right)P_{i}\tilde{x}\left(k_{0}\right)\\ ={\tilde{x}}^{T}\left(k_{0}\right)R_{s}^{1/2}{\bar{S}}_{i}^{-1}R_{s}^{1/2}\tilde{x}\left(k_{0}\right)\leq \frac{1}{\eta_{2}}{\tilde{x}}^{T}\left(k_{0}\right)R_{s}\tilde{x}\left(k_{0}\right) \end{array}$$

Moreover, using ${\tilde{x}}^{T}\left(k_{0}\right)R_{s}\tilde{x}\left(k_{0}\right)\leq c_{1}$ , we can obtain the following expression:


$$\begin{array}{c} {\tilde{x}}^{T}\left(k\right)R_{s}\tilde{x}\left(k\right)\leq \eta_{1}V_{\sigma \left(k\right)}\left(k\right)\leq \eta_{1}\left[\prod_{i=1}^{n}\mu_{i}^{N_{\sigma ,i}\left(k_{0},k\right)}{\tilde{a}}_{i}^{T_{i}\left(k_{0},k\right)}\hbar_{i}^{\Delta_{i}N_{\sigma ,i}\left(k_{0},k\right)}\right]\\ \left(\frac{c_{1}}{\eta_{2}}+\gamma^{2}{\tilde{b}}_{\text{max}}^{N_{f}}\eta_{3}\bar{\omega }\right)\\ \leq \exp\left\{\ln\eta_{1}+\sum_{i=1}^{n}\begin{array}{l} \left[\begin{array}{l} \frac{T_{i}\left(k_{0},k\right)}{\tau_{\mathrm{ai}}}\ln\mu_{i}+T_{i}\left(k_{0},k\right)\ln{\tilde{a}}_{i}\\ +\Delta_{i}\frac{T_{i}\left(k_{0},k\right)}{\tau_{\mathrm{ai}}}\ln\hbar_{i} \end{array}\right]\\ +\ln\left[\frac{c_{1}}{\eta_{2}}+\gamma^{2}{\tilde{b}}_{\text{max}}^{N_{f}}\eta_{3}\bar{\omega }\right] \end{array}\right\}\\ \leq \exp\left\{\ln\eta_{1}+\sum_{i=1}^{n}\begin{array}{l} \left[\frac{\ln\mu_{i}}{\tau_{\mathrm{ai}}}+\ln{\tilde{a}}_{i}+\frac{\Delta_{i}\ln\hbar_{i}}{\tau_{\mathrm{ai}}}\right]T_{i}\left(k_{0},k\right)\\ +\ln\left[\frac{c_{1}}{\eta_{2}}+\gamma^{2}{\tilde{b}}_{\text{max}}^{N_{f}}\eta_{3}\bar{\omega }\right] \end{array}\right\} \end{array}$$

Based on Definition 2, we have ${\tilde{x}}^{T}\left(k\right)R_{s}\tilde{x}\left(k\right)\leq c_{2}$ , which can be expressed as follows:


(37) $$\begin{array}{l} \exp\left\{\begin{array}{l} \ln\eta_{1}+\overset{n}{\underset{i=1}{\sum }}\left[\frac{\ln\mu_{i}}{\tau_{\mathrm{ai}}}+\ln{\tilde{a}}_{i}+\frac{\Delta_{i}\ln\hbar_{i}}{\tau_{\mathrm{ai}}}\right]\\ T_{i}\left(k_{0},k\right)+\ln\left[\frac{c_{1}}{\eta_{2}}+\gamma^{2}{\tilde{b}}_{\text{max}}^{N_{f}}\lambda_{3}\bar{\omega }\right] \end{array}\right\}\leq c_{2}\\ \Leftrightarrow \left(\frac{\ln\mu_{i}+\Delta_{i}\ln\hbar_{i}}{\tau_{\mathrm{ai}}}\right)N_{f}\leq \ln\frac{c_{2}}{\eta_{1}}-\ln\left[\frac{c_{1}}{\eta_{2}}+\gamma^{2}{\tilde{b}}_{\text{max}}^{N_{f}}\eta_{3}\bar{\omega }\right]-N_{f}\ln{\tilde{a}}_{i}\\ \Leftrightarrow \tau_{\mathrm{ai}}\geq \frac{N_{f}\ln\mu_{i}+N_{f}\Delta_{i}\ln\hbar_{i}}{\ln\frac{c_{2}}{\eta_{1}}-\ln\left[\frac{c_{1}}{\eta_{2}}+\gamma^{2}{\tilde{b}}_{\text{max}}^{N_{f}}\eta_{3}\bar{\omega }\right]-N_{f}\ln{\tilde{a}}_{i}} \end{array}$$

If Eqs. (23), (24) hold, then we can conclude that the following expression is true:


(38) $$\begin{array}{l} \ln\frac{c_{2}}{\eta_{1}}-\ln\left[\frac{c_{1}}{\eta_{2}}+\gamma^{2}{\tilde{b}}_{\text{max}}^{N_{f}}\eta_{3}\bar{\omega }\right]-N_{f}\ln{\tilde{a}}_{i}>0,\tau_{\mathrm{ai}}\\ \geq \frac{N_{f}\ln\mu_{i}+N_{f}\Delta_{i}\ln\hbar_{i}}{\ln\frac{c_{2}}{\eta_{1}}-\ln\left[\frac{c_{1}}{\eta_{2}}+\gamma^{2}{\tilde{b}}_{\text{max}}^{N_{f}}\eta_{3}\bar{\omega }\right]-N_{f}\ln{\tilde{a}}_{i}} \end{array}$$

which is equivalent to ${\tilde{x}}^{T}\left(k\right)R_{s}\tilde{x}\left(k\right)\leq c_{2}$ . Thus, the switched system in Eq. (12) is FTB, which completes the proof.

The sufficient guarantees of FTS are given in Theorem 1, and the prescribed attenuation performance are discussed in Theorem 2.

*Theorem 2*: Given system Eq. (12) and constant scalars $0<a_{i}<1$ , $b_{i}>0$ , $\mu_{i}\geq 1$ , γ>0 , if matrices $S_{i}>0$ , $S_{j}>0$ , $S_{ij}>0$ , and $W_{i}$ , ∀i,j∈Ω,i≠j , such that the following expression holds:


(39) $$S_{j}\leq \mu_{i}S_{i}$$

(40) $$\left[\begin{array}{cccccc} -S_{i} & 0 & 0 & 0 & {\tilde{A}}_{ii}S_{i} & {\tilde{B}}_{i}\\ {}^{*} & -S_{i} & 0 & 0 & \tilde{\rho }{\tilde{A}}_{1i}S_{i} & 0\\ {}^{*} & {}^{*} & -I & 0 & {\tilde{C}}_{i}S_{i} & {\tilde{D}}_{i}\\ {}^{*} & {}^{*} & {}^{*} & -I & \tilde{\rho }{\tilde{C}}_{1i}S_{i} & 0\\ {}^{*} & {}^{*} & {}^{*} & {}^{*} & -\left(1-a_{i}\right)S_{i} & 0\\ {}^{*} & {}^{*} & {}^{*} & {}^{*} & {}^{*} & -\gamma^{2}I \end{array}\right]<0$$

(41) $$\left[\begin{array}{cccccc} -S_{ij} & 0 & 0 & 0 & {\tilde{A}}_{ij}S_{j} & {\tilde{B}}_{i}\\ {}^{*} & -S_{ij} & 0 & 0 & \tilde{\rho }{\tilde{A}}_{1i}S_{j} & 0\\ {}^{*} & {}^{*} & -I & 0 & {\tilde{C}}_{i}S_{j} & {\tilde{D}}_{i}\\ {}^{*} & {}^{*} & {}^{*} & -I & \tilde{\rho }{\tilde{C}}_{1i}S_{j} & 0\\ {}^{*} & {}^{*} & {}^{*} & {}^{*} & -\left(1+b_{i}\right)\left(S_{ij}-S_{j}-S_{j}^{T}\right) & 0\\ {}^{*} & {}^{*} & {}^{*} & {}^{*} & {}^{*} & -\gamma^{2}I \end{array}\right]<0$$

then the system with MDADT satisfying the following expression is FTS with $H_{\infty }$ performance $\gamma_{d}$ with respect to $\left(0,c_{2},\bar{\omega },N_{f},R_{s},\gamma_{d}\right)$ .


(42) $$\tau_{\mathrm{ai}}\geq \tau_{\mathrm{ai}}^{*}=\max\left\{\begin{array}{l} \frac{N_{f}\ln\mu_{i}+N_{f}\Delta_{i}\ln\hbar_{i}}{\ln\frac{c_{2}}{\eta_{1}}-\ln\left[\gamma^{2}{\tilde{b}}_{\text{max}}^{N_{f}}\bar{\omega }\right]-N_{f}\ln{\tilde{a}}_{i}},\\ \frac{\Delta_{i}\ln\hbar_{i}+\ln\mu_{i}}{-\ln{\tilde{a}}_{i}} \end{array}\right\}$$

(43) $$\left(\gamma^{2}{\tilde{b}}_{\text{max}}^{N_{f}}\bar{\omega }\right){{\tilde{a}}_{i}}^{N_{f}}\leq \frac{c_{2}}{\eta_{1}}$$

where $\eta_{1}=\max_{i\in \Omega }\left(\lambda_{\text{max}}\left({\bar{S}}_{i}\right),\lambda_{\text{max}}\left({\bar{S}}_{ij}\right)\right)\text{,}$ $\tilde{\rho }=\sqrt{\rho \left(1-\rho \right)}\text{,}$ ${\bar{S}}_{i}=R_{s}^{1/2}S_{i}R_{s}^{1/2}\text{,}$ ${\bar{S}}_{j}=R_{s}^{1/2}S_{j}R_{s}^{1/2}\text{,}$ ${\tilde{a}}_{i}=1-a_{i}\text{,}$ ${\tilde{b}}_{i}=1+b_{i}\text{,}$ $\hbar_{i}={\tilde{b}}_{i}/{\tilde{a}}_{i}$ $\gamma_{d}=\gamma {\left(\frac{{\tilde{a}}_{\text{max}}}{{\tilde{a}}_{\text{min}}}\right)}^{N_{f}/2}\text{,}$ ${\tilde{a}}_{\text{max}}=\max\left\{{\tilde{a}}_{i}\right\}\text{,}$ ${\tilde{a}}_{\text{min}}=\min\left\{{\tilde{a}}_{i}\right\}\text{,}$ ${\tilde{b}}_{\text{max}}=\max\left\{{\tilde{b}}_{i}\right\}$ . Proof: The Lyapunov functions are determined in Eq. (25). We can obtain the following equations under the zero-initial condition.


(44) $$\begin{array}{l} \;\Delta V_{i}\left(k\right)+a_{i}V_{i}\left(k\right)+E\left\{e^{T}\left(k\right)e\left(k\right)\right\}-\gamma^{2}{\tilde{\omega }}^{T}\left(k\right)\tilde{\omega }\left(k\right)\\ =Ε\left\{\tilde{x}{\left(k+1\right)}^{T}P_{i}\tilde{x}\left(k+1\right)\right\}-\left(1-a_{i}\right){\tilde{x}}^{T}\left(k\right)P_{i}\tilde{x}\left(k\right)\\ \;+E\left\{e^{T}\left(k\right)e\left(k\right)\right\}-\gamma^{2}{\tilde{\omega }}^{T}\left(k\right)\tilde{\omega }\left(k\right)\\ =\xi^{T}\left(k\right)\{\begin{array}{l} \left[\begin{array}{c} {\tilde{A}}_{ii}^{T}\\ {\tilde{B}}_{i}^{T} \end{array}\right]P_{i}\left[\begin{array}{cc} {\tilde{A}}_{ii} & {\tilde{B}}_{i} \end{array}\right]+{\tilde{\rho }}^{2}\left[\begin{array}{c} {\tilde{A}}_{1i}^{T}\\ 0 \end{array}\right]P_{i}\left[\begin{array}{cc} {\tilde{A}}_{1i} & 0 \end{array}\right]\\ +\left[\begin{array}{c} {\tilde{C}}_{i}^{T}\\ {\tilde{D}}_{i}^{T} \end{array}\right]\left[\begin{array}{cc} {\tilde{C}}_{i} & {\tilde{D}}_{i} \end{array}\right] \end{array}\\ \;+{\tilde{\rho }}^{2}\left[\begin{array}{c} {\tilde{C}}_{1i}^{T}\\ 0 \end{array}\right]\left[\begin{array}{cc} {\tilde{C}}_{1i} & 0 \end{array}\right]+\left[\begin{array}{cc} -\left(1-a_{i}\right)P_{i} & 0\\ 0 & -\gamma^{2}I \end{array}\right]\}\xi \left(k\right)\\ =\xi^{T}\left(k\right)Z_{ii}\xi \left(k\right) \end{array}$$

(45) $$\begin{array}{l} \;\Delta V_{i}\left(k\right)-b_{i}V_{i}\left(k\right)+E\left\{e^{T}\left(k\right)e\left(k\right)\right\}-\gamma^{2}{\tilde{\omega }}^{T}\left(k\right)\tilde{\omega }\left(k\right)\\ =Ε\left\{\tilde{x}{\left(k+1\right)}^{T}P_{ij}\tilde{x}\left(k+1\right)\right\}-\left(1+b_{i}\right){\tilde{x}}^{T}\left(k\right)P_{ij}\tilde{x}\left(k\right)\\ \;+E\left\{e^{T}\left(k\right)e\left(k\right)\right\}-\gamma^{2}{\tilde{\omega }}^{T}\left(k\right)\tilde{\omega }\left(k\right)\\ =\xi^{T}\left(k\right)\{\begin{array}{l} \left[\begin{array}{c} {\tilde{A}}_{ij}^{T}\\ {\tilde{B}}_{i}^{T} \end{array}\right]P_{ij}\left[\begin{array}{cc} {\tilde{A}}_{ij} & {\tilde{B}}_{i} \end{array}\right]+{\tilde{\rho }}^{2}\left[\begin{array}{c} {\tilde{A}}_{1i}^{T}\\ 0 \end{array}\right]P_{ij}\left[\begin{array}{cc} {\tilde{A}}_{1i} & 0 \end{array}\right]\\ +\left[\begin{array}{c} {\tilde{C}}_{i}^{T}\\ {\tilde{D}}_{i}^{T} \end{array}\right]\left[\begin{array}{cc} {\tilde{C}}_{i} & {\tilde{D}}_{i} \end{array}\right] \end{array}\\ \;+{\tilde{\rho }}^{2}\left[\begin{array}{c} {\tilde{C}}_{1i}^{T}\\ 0 \end{array}\right]\left[\begin{array}{cc} {\tilde{C}}_{1i} & 0 \end{array}\right]+\left[\begin{array}{cc} -\left(1+b_{i}\right)P_{ij} & 0\\ 0 & -\gamma^{2}I \end{array}\right]\}\xi \left(k\right)\\ =\xi^{T}\left(k\right)Z_{ij}\xi \left(k\right) \end{array}$$

where


$$Z_{ii}=\left[\begin{array}{cccccc} -P_{i} & 0 & 0 & 0 & P_{i}{\tilde{A}}_{ii} & P_{i}{\tilde{B}}_{i}\\ {}^{*} & -P_{i} & 0 & 0 & \tilde{\rho }P_{i}{\tilde{A}}_{1i} & 0\\ {}^{*} & {}^{*} & -I & 0 & {\tilde{C}}_{i} & {\tilde{D}}_{i}\\ {}^{*} & {}^{*} & {}^{*} & -I & \tilde{\rho }{\tilde{C}}_{1i} & 0\\ {}^{*} & {}^{*} & {}^{*} & {}^{*} & -\left(1-a_{i}\right)P_{i} & 0\\ {}^{*} & {}^{*} & {}^{*} & {}^{*} & {}^{*} & -\gamma^{2}I \end{array}\right]\text{,}$$

$$Z_{ij}=\left[\begin{array}{cccccc} -P_{ij} & 0 & 0 & 0 & P_{ij}{\tilde{A}}_{ij} & P_{ij}{\tilde{B}}_{i}\\ {}^{*} & -P_{ij} & 0 & 0 & \tilde{\rho }P_{ij}{\tilde{A}}_{1i} & 0\\ {}^{*} & {}^{*} & -I & 0 & {\tilde{C}}_{i} & {\tilde{D}}_{i}\\ {}^{*} & {}^{*} & {}^{*} & -I & \tilde{\rho }{\tilde{C}}_{1i} & 0\\ {}^{*} & {}^{*} & {}^{*} & {}^{*} & -\left(1+b_{i}\right)P_{ij} & 0\\ {}^{*} & {}^{*} & {}^{*} & {}^{*} & {}^{*} & -\gamma^{2}I \end{array}\right]$$

The system in Eq. (12) is stable with predefined performance such that


(46) $$Z_{ii}<0$$

(47) $$Z_{ij}<0$$

Setting $S_{i}=P_{i}^{-1}$ and performing congruence transformation to the aforementioned inequalities through $\mathrm{diag}\left\{S_{i},S_{i},I,I,S_{i},I\right\}$ and $\mathrm{diag}\left\{S_{ij},S_{ij},I,I,S_{j},I\right\}$ , we can obtain the following expression:


(48) $$\left[\begin{array}{cccccc} -S_{i} & 0 & 0 & 0 & {\tilde{A}}_{ii}S_{i} & {\tilde{B}}_{i}\\ {}^{*} & -S_{i} & 0 & 0 & \tilde{\rho }{\tilde{A}}_{1i}S_{i} & 0\\ {}^{*} & {}^{*} & -I & 0 & {\tilde{C}}_{i}S_{i} & {\tilde{D}}_{i}\\ {}^{*} & {}^{*} & {}^{*} & -I & \tilde{\rho }{\tilde{C}}_{1i}S_{i} & 0\\ {}^{*} & {}^{*} & {}^{*} & {}^{*} & -\left(1-a_{i}\right)S_{i} & 0\\ {}^{*} & {}^{*} & {}^{*} & {}^{*} & {}^{*} & -\gamma^{2}I \end{array}\right]<0$$

(49) $$\left[\begin{array}{cccccc} -S_{i} & 0 & 0 & 0 & {\tilde{A}}_{ii}S_{i} & {\tilde{B}}_{i}\\ {}^{*} & -S_{i} & 0 & 0 & \tilde{\rho }{\tilde{A}}_{1i}S_{i} & 0\\ {}^{*} & {}^{*} & -I & 0 & {\tilde{C}}_{i}S_{i} & {\tilde{D}}_{i}\\ {}^{*} & {}^{*} & {}^{*} & -I & \tilde{\rho }{\tilde{C}}_{1i}S_{i} & 0\\ {}^{*} & {}^{*} & {}^{*} & {}^{*} & -\left(1-a_{i}\right)S_{i} & 0\\ {}^{*} & {}^{*} & {}^{*} & {}^{*} & {}^{*} & -\gamma^{2}I \end{array}\right]<0$$

Similar to the transformation in Eq. (33), we can obtain the following expression:


(50) $$S_{ij}-S_{j}-S_{j}^{T}\geq -S_{j}^{T}S_{ij}^{-1}S_{j}$$

With Eqs.(40), (41), we have $Z_{ii}<0$ and $Z_{ij}<0$ , which implies that the following expression:


(51) $$\Delta V_{i}\left(k\right)\leq \{\begin{array}{l} \begin{array}{l} -a_{i}V_{i}\left(k\right)-E\left\{e^{T}\left(k\right)e\left(k\right)\right\}+\gamma^{2}{\tilde{\omega }}^{T}\left(k\right)\tilde{\omega }\left(k\right),\\ \forall k\in [k_{i}+\Delta_{i},k_{i+1}) \end{array}\\ \begin{array}{l} b_{i}V_{i}\left(k\right)-E\left\{e^{T}\left(k\right)e\left(k\right)\right\}+\gamma^{2}{\tilde{\omega }}^{T}\left(k\right)\tilde{\omega }\left(k\right),\\ \forall k\in [k_{i},k_{i}+\Delta_{i}) \end{array} \end{array}$$

The following equation can be obtained by setting $\gamma^{2}{\tilde{\omega }}^{T}\left(k\right)W_{i}\tilde{\omega }\left(k\right)$ as $\psi \left(s\right)=-E\left\{e^{T}\left(k\right)e\left(k\right)\right\}+\gamma^{2}{\tilde{\omega }}^{T}\left(k\right)\tilde{\omega }\left(k\right)$ . Moreover, the system in Eq. (12) is FTB with respect to $\left(0,c_{2},\bar{\omega },N_{f},R_{s}\right)$ by setting $W_{i}=I$ and $c_{1}=0$ .


(52) $$\begin{array}{l} V_{i}\left(k\right)\leq \overset{n}{\underset{i=1}{\prod }}\mu_{i}^{N_{\sigma ,i}\left(k_{0},k\right)}{\tilde{a}}_{i}^{T_{i}\left(k_{0},k\right)}\hbar_{i}^{\Delta_{i}N_{\sigma ,i}\left(k_{0},k\right)}V_{\sigma \left(k_{0}\right)}\left(k_{0}\right)\\ +{\tilde{b}}_{\text{max}}^{N_{f}}\overset{k-1}{\underset{s=k_{0}}{\sum }}\left(\overset{n}{\underset{i=1}{\prod }}\mu_{i}^{N_{\sigma ,i}\left(s,k\right)}{\tilde{a}}_{i}^{T_{i}\left(s,k\right)}\hbar_{i}^{\Delta_{i}N_{\sigma ,i}\left(s,k\right)}\right)\psi \left(s\right) \end{array}$$

According to $V_{\sigma \left(k\right)}\left(k\right)\geq 0$ and zero-initial condition, we have the following expression:


(53) $$\begin{array}{l} \overset{k-1}{\underset{s=k_{0}}{\sum }}\left(\overset{n}{\underset{i=1}{\prod }}\mu_{i}^{N_{\sigma ,i}\left(s,k\right)}{\tilde{a}}_{i}^{T_{i}\left(s,k\right)}\hbar_{i}^{\Delta_{i}N_{\sigma ,i}\left(s,k\right)}\right)\psi \left(s\right)\geq 0\\ \Leftrightarrow \overset{k-1}{\underset{s=k_{0}}{\sum }}\left(\overset{n}{\underset{i=1}{\prod }}\mu_{i}^{N_{\sigma ,i}\left(s,k\right)}{\tilde{a}}_{i}^{T_{i}\left(s,k\right)}\hbar_{i}^{\Delta_{i}N_{\sigma ,i}\left(s,k\right)}\right)e^{T}\left(k\right)e\left(k\right)\\ \leq \gamma^{2}\overset{k-1}{\underset{s=k_{0}}{\sum }}\left(\overset{n}{\underset{i=1}{\prod }}\mu_{i}^{N_{\sigma ,i}\left(s,k\right)}{\tilde{a}}_{i}^{T_{i}\left(s,k\right)}\hbar_{i}^{\Delta_{i}N_{\sigma ,i}\left(s,k\right)}\right){\tilde{\omega }}^{T}\left(s\right)\tilde{\omega }\left(s\right) \end{array}$$

Multiplying both sides of Eq. (53) by $\overset{n}{\underset{i=1}{\prod }}{\left(\hbar_{i}^{\Delta_{i}}\mu_{i}\right)}^{-N_{\sigma i}\left(k_{0},k\right)}$ , we obtain the following equation:


(54) $$\begin{array}{l} \overset{k-1}{\underset{s=k_{0}}{\sum }}\left(\overset{n}{\underset{i=1}{\prod }}\mu_{i}^{-N_{\sigma ,i}\left(k_{0},s\right)}{\tilde{a}}_{i}^{T_{i}\left(s,k\right)}\hbar_{i}^{-\Delta_{i}N_{\sigma ,i}\left(k_{0},s\right)}\right)e^{T}\left(k\right)e\left(k\right)\\ \leq \gamma^{2}\overset{k-1}{\underset{s=k_{0}}{\sum }}\left(\overset{n}{\underset{i=1}{\prod }}\mu_{i}^{-N_{\sigma ,i}\left(k_{0},s\right)}{\tilde{a}}_{i}^{T_{i}\left(s,k\right)}\hbar_{i}^{-\Delta_{i}N_{\sigma ,i}\left(k_{0},s\right)}\right){\tilde{\omega }}^{T}\left(s\right)\tilde{\omega }\left(s\right) \end{array}$$

Based on the definition of MDADT and Eq. (42), we have the following:


(55) $$0\leq N_{\sigma ,i}\left(k_{0},s\right)\leq \frac{T_{i}\left(k_{0},s\right)}{\tau_{\mathrm{ai}}^{*}}\leq \frac{-T_{i}\left(k_{0},s\right)\ln{\tilde{a}}_{i}}{\Delta_{i}\left(\ln{\tilde{b}}_{i}-\ln{\tilde{a}}_{i}\right)+\ln\mu_{i}}$$

Combining with Eqs. (43), (45), we infer the following:


(56) $$\begin{array}{l} \overset{k-1}{\underset{s=k_{0}}{\sum }}\left(\overset{n}{\underset{i=1}{\prod }}{\left(\mu_{i}\hbar_{i}^{\Delta_{i}}\right)}^{\frac{T_{i}\left(k_{0},s\right)\ln{\tilde{a}}_{i}}{\Delta_{i}\left(\ln{\tilde{b}}_{i}-\ln{\tilde{a}}_{i}\right)+\ln\mu_{i}}}{\tilde{a}}_{i}^{T_{i}\left(s,k\right)}\right)e^{T}\left(k\right)e\left(k\right)\\ \leq \gamma^{2}\overset{k-1}{\underset{s=k_{0}}{\sum }}\left(\overset{n}{\underset{i=1}{\prod }}{\left(\mu_{i}\hbar_{i}^{\Delta_{i}}\right)}^{\frac{T_{i}\left(k_{0},s\right)\ln{\tilde{a}}_{i}}{\Delta_{i}\left(\ln{\tilde{b}}_{i}-\ln{\tilde{a}}_{i}\right)+\ln\mu_{i}}}{\tilde{a}}_{i}^{T_{i}\left(s,k\right)}\right){\tilde{\omega }}^{T}\left(s\right)\tilde{\omega }\left(s\right) \end{array}$$

Thus, we have the following equation:


(57) $${\left(\mu_{i}\hbar_{i}^{\Delta_{i}}\right)}^{\frac{T_{i}\left(k_{0},s\right)\ln{\tilde{a}}_{i}}{\Delta_{i}\left(\ln{\tilde{b}}_{i}-\ln{\tilde{a}}_{i}\right)+\ln\mu_{i}}}={\tilde{a}}_{i}^{T_{i}\left(k_{0},s\right)}$$

Next, we have the following expression:


(58) $$\overset{k-1}{\underset{s=k_{0}}{\sum }}\left(\overset{n}{\underset{i=1}{\prod }}{\tilde{a}}_{i}^{T_{i}\left(k_{0},k\right)}\right)e^{T}\left(k\right)e\left(k\right)\leq \gamma^{2}\overset{k-1}{\underset{s=k_{0}}{\sum }}\left(\overset{n}{\underset{i=1}{\prod }}{\tilde{a}}_{i}^{T_{i}\left(k_{0},k\right)}\right){\tilde{\omega }}^{T}\left(s\right)\tilde{\omega }\left(s\right)$$

Setting $k-1=N_{f}$ , we can obtain the following:


$$\begin{array}{l} \overset{N_{f}}{\underset{s=k_{0}}{\sum }}\left(\overset{n}{\underset{i=1}{\prod }}{\tilde{a}}_{i}^{T_{i}\left(k_{0},k\right)}\right)e^{T}\left(k\right)e\left(k\right)\leq \gamma^{2}\overset{k-1}{\underset{s=k_{0}}{\sum }}\left(\overset{n}{\underset{i=1}{\prod }}{\tilde{a}}_{i}^{T_{i}\left(k_{0},k\right)}\right){\tilde{\omega }}^{T}\left(s\right)\tilde{\omega }\left(s\right)\\ \Leftrightarrow {\tilde{a}}_{\text{min}}^{N_{f}}\overset{N_{f}}{\underset{s=k_{0}}{\sum }}e^{T}\left(k\right)e\left(k\right)\leq \gamma^{2}{\tilde{a}}_{\text{max}}^{N_{f}}\overset{k-1}{\underset{s=k_{0}}{\sum }}{\tilde{\omega }}^{T}\left(s\right)\tilde{\omega }\left(s\right)\\ \Leftrightarrow \overset{N_{f}}{\underset{s=k_{0}}{\sum }}e^{T}\left(k\right)e\left(k\right)\leq \gamma^{2}{\left(\frac{{\tilde{a}}_{\text{max}}}{{\tilde{a}}_{\text{min}}}\right)}^{N_{f}}\overset{k-1}{\underset{s=k_{0}}{\sum }}{\tilde{\omega }}^{T}\left(s\right)\tilde{\omega }\left(s\right) \end{array}$$

Therefore, the system Eq. (12) is FTB with given attenuation index $\gamma_{d}=\gamma {\left(\frac{{\tilde{a}}_{\text{max}}}{{\tilde{a}}_{\text{min}}}\right)}^{N_{f}/2}$ , which completes the proof.

Based on Theorems 1 and 2, the parameters of finite-time tracking controller of switched systems is derived in Theorem 3.

*Theorem 3*: Given system Eq. (12) and constant scalars $0<a_{i}<1$ , $b_{i}>0$ , $\mu_{i}\geq 1$ , γ>0 , if positive matrices $S_{i}$ , $S_{j}$ and $S_{ij}$ , ∀i,j∈Ω,i≠j , exist such that the following holds true:


(59) $$S_{j}\leq \mu_{i}S_{i}$$

(60) $$\left[\begin{array}{ccc} \Phi_{ii} & \Sigma_{1i} & \Sigma_{2i}\\ {}^{*} & \Xi_{1i} & 0\\ {}^{*} & {}^{*} & \Xi_{2i} \end{array}\right]<0$$

(61) $$\left[\begin{array}{ccc} \Phi_{ij} & \Sigma_{1j} & \Sigma_{2j}\\ {}^{*} & \Xi_{1j} & 0\\ {}^{*} & {}^{*} & \Xi_{2j} \end{array}\right]<0$$

System Eq. (12) with MDADT satisfying Eqs. (42), (43) is finite-time stable with predefined attenuation index $\gamma_{d}$ with respect to $\left(0,c_{2},\bar{\omega },N_{f},R_{s},\gamma_{d}\right)$ , and the parameters of robust controller can be expressed as follows:


(62) $$K_{n,1i}=U_{1i}S_{1i}^{-1}$$

(63) $$K_{n,2i}=U_{2i}S_{2i}^{-1}$$

where


$$S_{i}=\left[\begin{array}{cc} S_{1i} & 0\\ 0 & S_{2i} \end{array}\right]\text{,}$$

$$S_{j}=\left[\begin{array}{cc} S_{1j} & 0\\ 0 & S_{2j} \end{array}\right]$$

$$\Phi_{ii}=\left[\begin{array}{cccccc} -S_{i} & 0 & 0 & 0 & \varphi_{ii} & {\tilde{B}}_{i}\\ {}^{*} & -S_{i} & 0 & 0 & \tilde{\rho }{\tilde{A}}_{1i}S_{i} & 0\\ {}^{*} & {}^{*} & -I & 0 & {\tilde{C}}_{i}S_{i} & {\tilde{D}}_{i}\\ {}^{*} & {}^{*} & {}^{*} & -I & \tilde{\rho }{\tilde{C}}_{1i}S_{i} & 0\\ {}^{*} & {}^{*} & {}^{*} & {}^{*} & -\left(1-a_{i}\right)S_{i} & 0\\ {}^{*} & {}^{*} & {}^{*} & {}^{*} & {}^{*} & -\gamma^{2}I \end{array}\right]\text{,}$$

$$\Phi_{ij}=\left[\begin{array}{cccccc} -S_{ij} & 0 & 0 & 0 & \varphi_{ij} & {\tilde{B}}_{i}\\ {}^{*} & -S_{ij} & 0 & 0 & \tilde{\rho }{\tilde{A}}_{1i}S_{j} & 0\\ {}^{*} & {}^{*} & -I & 0 & {\tilde{C}}_{i}S_{j} & {\tilde{D}}_{i}\\ {}^{*} & {}^{*} & {}^{*} & -I & \tilde{\rho }{\tilde{C}}_{1i}S_{j} & 0\\ {}^{*} & {}^{*} & {}^{*} & {}^{*} & -\left(1+b_{i}\right)\left(S_{ij}-S_{j}-S_{j}^{T}\right) & 0\\ {}^{*} & {}^{*} & {}^{*} & {}^{*} & {}^{*} & -\gamma^{2}I \end{array}\right]\text{,}$$

$$\Sigma_{1i}=\left[\begin{array}{cc} {\tilde{M}}_{1i}^{T} & {\tilde{N}}_{1i}^{T} \end{array}\right]\text{,}$$

$$\Sigma_{2i}=\left[\begin{array}{cc} {\tilde{M}}_{2i}^{T} & {\tilde{N}}_{2i}^{T} \end{array}\right]\text{,}$$

$$\Sigma_{1j}=\left[\begin{array}{cc} {\tilde{M}}_{1j}^{T} & {\tilde{N}}_{1j}^{T} \end{array}\right]\text{,}$$

$$\Sigma_{2j}=\left[\begin{array}{cc} {\tilde{M}}_{2j}^{T} & {\tilde{N}}_{2j}^{T} \end{array}\right]\text{,}$$

$$\Xi_{1i}=\mathrm{diag}\left\{\varepsilon_{1i},\varepsilon_{1i}\right\}\text{,}$$

$$\Xi_{2i}=\mathrm{diag}\left\{\varepsilon_{2i},\varepsilon_{2i}\right\}\text{,}$$

$$\Xi_{1j}=\mathrm{diag}\left\{\varepsilon_{1j},\varepsilon_{1j}\right\}\text{,}$$

$$\Xi_{2j}=\mathrm{diag}\left\{\varepsilon_{2j},\varepsilon_{2j}\right\}\text{,}$$

$$\varphi_{ii}=\left[\begin{array}{cc} A_{i}S_{1i}+B_{i}U_{1i} & B_{i}U_{2i}\\ -\rho C_{i}S_{1i} & S_{2i} \end{array}\right]\text{,}$$

$$\varphi_{ij}=\left[\begin{array}{cc} A_{i}S_{2j}+B_{i}U_{1j} & B_{i}U_{2j}\\ -\rho C_{i}S_{1j} & S_{2j} \end{array}\right]\text{,}$$

$${\tilde{M}}_{1i}=\left[\begin{array}{cccccccccc} M_{1i}^{T}{\tilde{B}}_{i}^{T} & 0 & 0 & 0 & 0 & 0 & 0 & 0 & 0 & 0 \end{array}\right]\text{,}$$

$${\tilde{N}}_{1i}=\left[\begin{array}{cccccccccc} 0 & 0 & 0 & 0 & 0 & 0 & N_{1i}S_{1i} & 0 & 0 & 0 \end{array}\right]\text{,}$$

$${\tilde{M}}_{2i}=\left[\begin{array}{cccccccccc} M_{2i}^{T}{\tilde{B}}_{i}^{T} & 0 & 0 & 0 & 0 & 0 & 0 & 0 & 0 & 0 \end{array}\right]\text{,}$$

$${\tilde{N}}_{2i}=\left[\begin{array}{cccccccccc} 0 & 0 & 0 & 0 & 0 & 0 & 0 & N_{2i}S_{2i} & 0 & 0 \end{array}\right]\text{,}$$

$${\tilde{M}}_{1j}=\left[\begin{array}{cccccccccc} M_{1j}^{T}{\tilde{B}}_{i}^{T} & 0 & 0 & 0 & 0 & 0 & 0 & 0 & 0 & 0 \end{array}\right]\text{,}$$

$${\tilde{N}}_{1j}=\left[\begin{array}{cccccccccc} 0 & 0 & 0 & 0 & 0 & 0 & N_{1j}S_{1j} & 0 & 0 & 0 \end{array}\right]\text{,}$$

$${\tilde{M}}_{2j}=\left[\begin{array}{cccccccccc} M_{2j}^{T}{\tilde{B}}_{i}^{T} & 0 & 0 & 0 & 0 & 0 & 0 & 0 & 0 & 0 \end{array}\right]\text{,}$$

$${\tilde{N}}_{2j}=\left[\begin{array}{cccccccccc} 0 & 0 & 0 & 0 & 0 & 0 & 0 & N_{2j}S_{2j} & 0 & 0 \end{array}\right]\text{,}$$

$$\varepsilon_{1i}>0\text{,}$$

$$\varepsilon_{2i}>0\text{,}$$

$$\varepsilon_{2j}>0.$$

Proof: According to Schur Complement (Aristidou et al., 2014) and Lemma 1, we can calculate the following equation:


(64) $$\Phi_{ii}+{\tilde{M}}_{1i}^{T}F_{1i}{\tilde{N}}_{1i}+{\tilde{N}}_{1i}^{T}F_{1i}^{T}{\tilde{M}}_{1i}+{\tilde{M}}_{2i}^{T}F_{2i}{\tilde{N}}_{2i}+{\tilde{N}}_{2i}^{T}F_{2i}^{T}{\tilde{M}}_{2i}<0$$

(65) $$\Phi_{ij}+{\tilde{M}}_{1j}^{T}F_{1j}{\tilde{N}}_{1j}+{\tilde{N}}_{1j}^{T}F_{1j}^{T}{\tilde{M}}_{1j}+{\tilde{M}}_{2j}^{T}F_{2j}{\tilde{N}}_{2j}+{\tilde{N}}_{2j}^{T}F_{2j}^{T}{\tilde{M}}_{2j}<0$$

Let $U_{1i}=K_{n,1i}S_{1i}$ , $U_{2i}=K_{n,2i}S_{2i}$ , $U_{1j}=K_{n,1j}S_{1j}$ , $U_{2j}=K_{n,2j}S_{2j}$ , Eq. (60) is equivalent to Eq. (40), and Eq. (61) is equivalent to Eq. (41). Therefore, the parameters of controller can be given according to Eqs. (59)–(61) by solving linear matrix inequalities Eqs. (62), (63).

### 3.2 Online scheduling based on the ADDPG algorithm

Based on the finite-time *H*_∞_ control, the sufficient conditions to ensure the FTS and prescribed performance are presented. The process of online scheduling can be formulated as the Markov decision process (MDP). Because the control process is a series of continuous decision process, the ADDPG algorithm was proposed based on the actor–critic framework to realize superior control performance of switched flight vehicles.

The DRL is composed of an agent and the interacting environment. At each time, the agent obtains a state $s_{k}$ , selects an action $a_{k}$ , and can receive reward $r_{k}$ and $s_{k+1}$ by interacting with the environment, in which $r_{k}$ is used to evaluate the performance of state-action pair at the time instant. In this study, the switched tracking controller can be viewed as the agent, whose purpose is maximizing the sum of the expected discounted reward function over a series of future steps:


(66) $$R\left(k\right)=r_{k}+\gamma_{d}r_{k+1}+\gamma_{d}^{2}r_{k+2}+\cdots +\gamma_{d}^{K_{f}-k}r_{K_{f}}=r_{k}+\gamma_{d}R_{k+1}$$

where $\gamma_{d}\in \left[0,1\right]$ denotes the discount factor. Here, $K_{f}$ denotes the terminal step of reinforcement learning. The value of reward depends on the action undertaken and the current state. The action and state are defined as follows:


(67) $$a_{k}=\left[\begin{array}{cc} \Delta K_{c,1i}\left(k\right) & \Delta K_{c,2i}\left(k\right) \end{array}\right]$$

(68) $$s_{k}=\left[\begin{array}{cccc} \alpha \left(k\right) & q\left(k\right) & r_{c}\left(k\right) & u_{n}\left(k\right) \end{array}\right]$$

The ADDPG algorithm is provided based on the DDPG algorithm, in which the advantages of both deep Q learning and actor–critic framework are used to realize the optimal action, which is updated in continuous action spaces based on policy gradient theory. The ADDPG algorithm is realized in the following two sections: the action-value in each step is approximated by the critic network $Q\left(s_{k},a_{k}|\varsigma^{Q}\right)$ with weights $\varsigma^{Q}$ , the current control policy is obtained by the actor network $\varpi \left(s_{k}|\varsigma^{\varpi }\right)$ with weights $\varsigma^{\varpi }$ . The weights of the critic network are updated by minimizing the loss function, which can be described as follows:


(69) $$L\left(\varsigma^{Q}\right)=E_{\left(s,a\right)}\left[{\left(Q\left(s_{k},a_{k}|\varsigma^{Q}\right)-{\bar{y}}_{k}\right)}^{2}\right]$$

where


$${\bar{y}}_{k}=r_{k}\left(s_{k},a_{k}\right)+\gamma_{d}Q\left(s_{k+1},\varpi \left(s_{k}|\varsigma^{\varpi }\right)|\varsigma^{Q}\right)\text{.}$$

The weights of actor network are updated according to the policy gradient in the following equations:


(70) $$\varsigma^{\varpi }\left(k+1\right)=\varsigma^{\varpi }\left(k\right)+L_{\mathrm{an}}\nabla_{\varsigma^{\varpi }}J$$

(71) $$\begin{array}{l} \nabla_{\varsigma^{\varpi }}J=E_{\pi }\left[{\nabla_{\varsigma^{\varpi }}Q^{\pi }\left(s_{k},\pi \left(s_{k}|\varsigma^{\varpi }\right)|\varsigma^{Q}\right)|}_{s=s_{k},a=\pi \left(s_{k}|\varsigma^{\varpi }\right)}\right]\\ \;=E_{\pi }\left[\nabla_{\varsigma^{\varpi }}Q^{\pi }\left(s_{k},\pi \left(s_{k}\right)|\varsigma^{Q}|\right)\nabla_{\varsigma^{\varpi }}\pi \left(s_{k}|\varsigma^{Q}\right)\right] \end{array}$$

where $L_{\mathrm{an}}$ is the learning rate of $\varpi \left(s_{k}|\varsigma^{\varpi }\right)$ .

To overcome the divergence of Q learning, two separated networks were adopted: the actor target network $\varpi^{\prime }\left(s_{k}|\varsigma^{\varpi '}\right)$ and the critic target network $Q^{\prime }\left(s_{k},a_{k}|\varsigma^{Q'}\right)$ , the mentioned two networks can update their weights as follows:


(72) $$\varsigma^{\varpi '}\left(k+1\right)=L_{atn}\varsigma^{\varpi }\left(k\right)+\left(1-L_{atn}\right)\varsigma^{\varpi '}\left(k\right)$$

(73) $$\varsigma^{Q'}\left(k+1\right)=L_{ctn}\varsigma^{Q}\left(k+1\right)+\left(1-L_{ctn}\right)\varsigma^{Q'}\left(k+1\right)$$

where $L_{atn}$ and $L_{ctn}$ are the learning rates.

Moreover, an exploration noise $N_{a}$ is added to the actor to realize exploration and actual control policy, which is generated by actor and can be rewritten as follows:


(74) $$a_{k}=\pi \left(s_{k}|\varsigma^{\varpi }\right)+N_{a}$$

Unlike the conventional DDPG algorithm, the adaptive parameters were introduced to achieve superior convergence and robustness, respectively. By introducing robustness as a continuous parameter, the reward function enables the convenient exploration to realize adaptive training. The control policy is used to reduce the tracking error with lower control input and unsaturated actuator, therefore, the reward function depends on the tracking error, amplitude of control signal, and the saturation of actuator, which can be expressed as follows:


(75) $$r_{k}=g_{1}r_{e1}\left(k\right)+g_{2}r_{e2}\left(k\right)+g_{3}r_{e3}\left(k\right)$$

(76) $$r_{e1}=\frac{\left|\upsilon_{1}-2\right|}{\upsilon_{1}}\left({\left(\frac{\left(e\left(k\right)/l_{1}\right)}{\left|\upsilon_{1}-2\right|}+1\right)}^{\frac{\upsilon_{1}}{2}}-1\right)$$

(77) $$r_{e2}=\frac{\left|\upsilon_{2}-2\right|}{\upsilon_{2}}\left({\left(\frac{\left(u\left(k\right)/l_{2}\right)}{\left|\upsilon_{2}-2\right|}+1\right)}^{\frac{\upsilon_{2}}{2}}-1\right)$$

(78) $$r_{e3}=\{\begin{array}{l} \delta_{p},\left|u\left(k\right)\right|>\bar{u}\left(k\right)\\ 0,\left|u\left(k\right)\right|\leq \bar{u}\left(k\right) \end{array}$$

where $r_{e1}\left(k\right)$ represents the reward of tracking error, $r_{e2}\left(k\right)$ denotes the reward of control input, and $r_{e3}\left(k\right)$ is the reward of saturation, respectively. Here, $g_{1}$ , $g_{2}$ , and $g_{3}$ denote the weights of $r_{e1}\left(k\right)$ , $r_{e2}\left(k\right)$ , and $r_{e3}\left(k\right)$ in the reward function. Furthermore, $\upsilon_{1}$ , $\upsilon_{2}$ are the adaptive shape parameters, which determine the robustness of the reward function. $l_{1}>0$ and $l_{2}>0$ are the parameters that controls the size of the quadratic bowl near the origin, respectively. Here, $\delta_{p}$ is predefined constant and $\bar{u}\left(k\right)$ denotes the upper bound of the actuator. Next, the final reward function $r_{e1}\left(k\right)$ and $r_{e2}\left(k\right)$ with adaptive parameters can be rewritten as follows:


(79) $$r_{e1}\left(k\right)=\{\begin{array}{l} \frac{1}{2}{\left(e\left(k\right)/l_{1}\right)}^{2}\;\mathrm{if}\;\upsilon_{1}=2\\ \log\left(\frac{1}{2}{\left(e\left(k\right)/l_{1}\right)}^{2}+1\right)\;\mathrm{if}\;\upsilon_{1}=0\\ 1-\exp\left(-\frac{1}{2}{\left(e\left(k\right)/l_{1}\right)}^{2}\right)\;\mathrm{if}\;\upsilon_{1}=-\infty \\ \frac{\left|\upsilon_{1}-2\right|}{\upsilon_{1}}\left({\left(\frac{\left(e\left(k\right)/l_{1}\right)}{\left|\upsilon_{1}-2\right|}+1\right)}^{\frac{\upsilon_{1}}{2}}-1\right)\;\mathrm{otherwise} \end{array}$$

(80) $$r_{e2}\left(k\right)=\{\begin{array}{l} \frac{1}{2}{\left(u\left(k\right)/l_{2}\right)}^{2}\;\mathrm{if}\;\upsilon_{2}=2\\ \log\left(\frac{1}{2}{\left(u\left(k\right)/l_{2}\right)}^{2}+1\right)\;\mathrm{if}\;\upsilon_{2}=0\\ 1-\exp\left(-\frac{1}{2}{\left(u\left(k\right)/l_{2}\right)}^{2}\right)\;\mathrm{if}\;\upsilon_{2}=-\infty \\ \frac{\left|\upsilon_{2}-2\right|}{\upsilon_{2}}\left({\left(\frac{\left(u\left(k\right)/l_{2}\right)}{\left|\upsilon_{2}-2\right|}+1\right)}^{\frac{\upsilon_{2}}{2}}-1\right)\;\mathrm{otherwise} \end{array}$$

The adaptive updating law of hyper parameters are defined as follows to improve transient performance and robustness of the algorithm:


(81) $$\left\{ \begin{array}{l} v_{1}^{\left(s\right)}=\left(v_{1\max}-v_{1\min}\right)\mathrm{sigmoid}\left(v_{1p}^{\left(s\right)}\right)+v_{1\min}\\ v_{2}^{\left(s\right)}=\left(v_{2\max}-v_{2\min}\right)\mathrm{sigmoid}\left(v_{2p}^{\left(s\right)}\right)+v_{2\min} \end{array}\right.$$

(82) $$\left\{ \begin{array}{l} l_{1}^{\left(s\right)}=\mathrm{softplus}\left(l_{1p}^{\left(s\right)}\right)+l_{1\min}\\ l_{2}^{\left(s\right)}=\mathrm{softplus}\left(l_{2p}^{\left(s\right)}\right)+l_{2\min} \end{array}\right.$$

where $v_{1\max}$ and $v_{1\min}$ denote the maximum and minimum values of $v_{1\max}$ . Similarly, we can obtain the definitions of $v_{2\max}$ , $v_{2\min}$ , $l_{1\min}$ , and $l_{2\min}$ . The length of each segment is determined by training episodes.

Based on the statement, the pseudocode for the ADDPG algorithm proposed in this paper is presented in Algorithm 1.

**Table:** 

Algorithm 1. Parameter optimization based on ADDPG
Set the variation range of controller parameters.
Design the switched tracking controllers for flight vehicles based on Theorem 3.
Randomly initialize the weights of networks $Q\left(s_{k},a_{k}|\varsigma^{Q}\right)$ and $\varpi \left(s_{k}|\varsigma^{\varpi }\right)$ with $\varsigma^{\varpi }$ and $\varsigma^{Q}$ .
Initialize the weights of $\varpi^{\prime }\left(s_{k}|\varsigma^{\varpi '}\right)$ and $Q^{\prime }\left(s_{k},a_{k}|\varsigma^{Q'}\right)$ with weights $\varsigma^{\varpi '}\leftarrow \varsigma^{\varpi }$ , $\varsigma^{Q'}\leftarrow \varsigma^{Q}$ .
Initialize the replay buffer, *episode* = 0
**for** *episode* = 1 to *M* do
Randomly initialize exploration noise $N_{a}$ .
Randomly initialize the state vector of the agent with *s*_1_, then the initial observation can be obtained.
**for** *t* = 1 to *K* do
Apply action $a_{k}=\pi \left(s_{k}|\varsigma^{\varpi }\right)+N_{a}$ to the environment based on the state $s_{k}$ and uncertain noise.
Receive the adaptive reward $r_{k}$ and the state of next time instant $s_{k+1}$ .
Store the variable transition pair in the replay buffer, which consists of $s_{k}$ , $a_{k}$ , $r_{k}$ , and $s_{k+1}$ .
Randomly sample a mini-batch of *N* transition pairs from the replay buffer R .
Set $y_{k}=r_{k}\left(s_{k},a_{k}\right)+\gamma_{p}Q\left(s_{k+1},\varpi \left(s_{k}|\varsigma^{\varpi }\right)|\varsigma^{Q}\right)$
Update the weights of network $Q\left(s_{k},a_{k}|\varsigma^{Q}\right)$ as follows: $L\left(\varsigma^{Q}\right)=E_{\left(s,a\right)}\left[{\left(Q\left(s_{k},a_{k}|\varsigma^{Q}\right)-y_{k}\right)}^{2}\right]$
Update the weights of network $\varpi \left(s_{k}|\varsigma^{\varpi }\right)$ as follows: $\nabla_{\varsigma^{\varpi }}J=E_{\pi }\left[\nabla_{\varsigma^{\varpi }}Q^{\pi }\left(s_{k},\pi \left(s_{k}\right)|\varsigma^{Q}|\right)\nabla_{\varsigma^{\varpi }}\pi \left(s_{k}|\varsigma^{Q}\right)\right]$
Update the weights of target networks: $\varsigma^{\varpi '}\leftarrow L_{atn}\varsigma^{\varpi }+\left(1-L_{atn}\right)\varsigma^{\varpi '}$ , $\varsigma^{Q'}\leftarrow L_{ctn}\varsigma^{Q}+\left(1-L_{ctn}\right)\varsigma^{Q'}$
**end for**
**end for**

*Remark 3*: Although the conventional DDPG algorithm can realize parameter optimization (Xu et al., 2019; Gaudet et al., 2020; Gheisarnejad and Khooban, 2021), guaranteeing data efficiency and system stability because it attempts to explore the optimal control policy for all possible action in the action space is difficult. Moreover, the proposed adaptive hyper parameters can increase robustness and achieve generalized case because the reward function determines training performance.

## 4 Numerical examples

In this study, the HiMAT vehicle is given to validate the proposed method. The three-view drawing and trim condition for operation points can be obtained from the study performed by Wang et al. (2015). The flight condition and the model of longitudinal motion dynamics are given as Wang et al. (2015).

Based on the trim condition within the flight envelope, the longitudinal motion dynamics can be described by switched systems. We set the sampling time $T_{s}=0.02$ and obtain the system matrices $A_{i}$ and $B_{i}$ , which can be described as follows:


$$\begin{array}{l} A_{1}=\left[\begin{array}{cc} 0.9804 & 0.0188\\ 0.1768 & 0.9720 \end{array}\right],B_{1}=\left[\begin{array}{ccc} -0.0049 & -0.0034 & 0.0007\\ -0.1579 & -0.0979 & 0.0993 \end{array}\right]\\ A_{2}=\left[\begin{array}{cc} 0.9728 & 0.0188\\ 0.3773 & 0.9622 \end{array}\right],B_{2}=\left[\begin{array}{ccc} -0.0075 & -0.0050 & 0.0014\\ -0.2941 & -0.1765 & 0.1831 \end{array}\right]\\ A_{4}=\left[\begin{array}{cc} 0.9688 & 0.0187\\ 0.4968 & 0.9560 \end{array}\right],B_{4}=\left[\begin{array}{ccc} -0.0096 & -0.0065 & 0.0021\\ -0.4334 & -0.2895 & 0.2547 \end{array}\right]\\ A_{8}=\left[\begin{array}{cc} 0.9766 & 0.0190\\ 0.3312 & 0.9668 \end{array}\right],B_{8}=\left[\begin{array}{ccc} -0.0077 & -0.0054 & 0.0018\\ -0.3759 & -0.2798 & 0.2113 \end{array}\right]\\ A_{9}=\left[\begin{array}{cc} 0.9725 & 0.0189\\ 0.3344 & 0.9594 \end{array}\right],B_{9}=\left[\begin{array}{ccc} -0.0099 & -0.0068 & 0.0026\\ -0.5374 & -0.3793 & 0.2890 \end{array}\right]\\ A_{12}=\left[\begin{array}{cc} 0.9649 & 0.0188\\ 0.2242 & 0.9509 \end{array}\right],B_{12}=\left[\begin{array}{ccc} -0.0136 & -0.0094 & 0.0042\\ -0.9015 & -0.6166 & 0.4367 \end{array}\right]\\ A_{18}=\left[\begin{array}{cc} 0.9657 & 0.0191\\ -0.9772 & 0.9523 \end{array}\right],B_{18}=\left[\begin{array}{ccc} -0.0061 & -0.0033 & 0.0023\\ -0.4595 & -0.2426 & 0.2576 \end{array}\right]\\ A_{19}=\left[\begin{array}{cc} 0.9635 & 0.0192\\ -1.2369 & 0.9507 \end{array}\right],B_{19}=\left[\begin{array}{ccc} -0.0066 & -0.0032 & 0.0019\\ -0.5334 & -0.2569 & 0.2163 \end{array}\right] \end{array}$$

**Figure 2:**
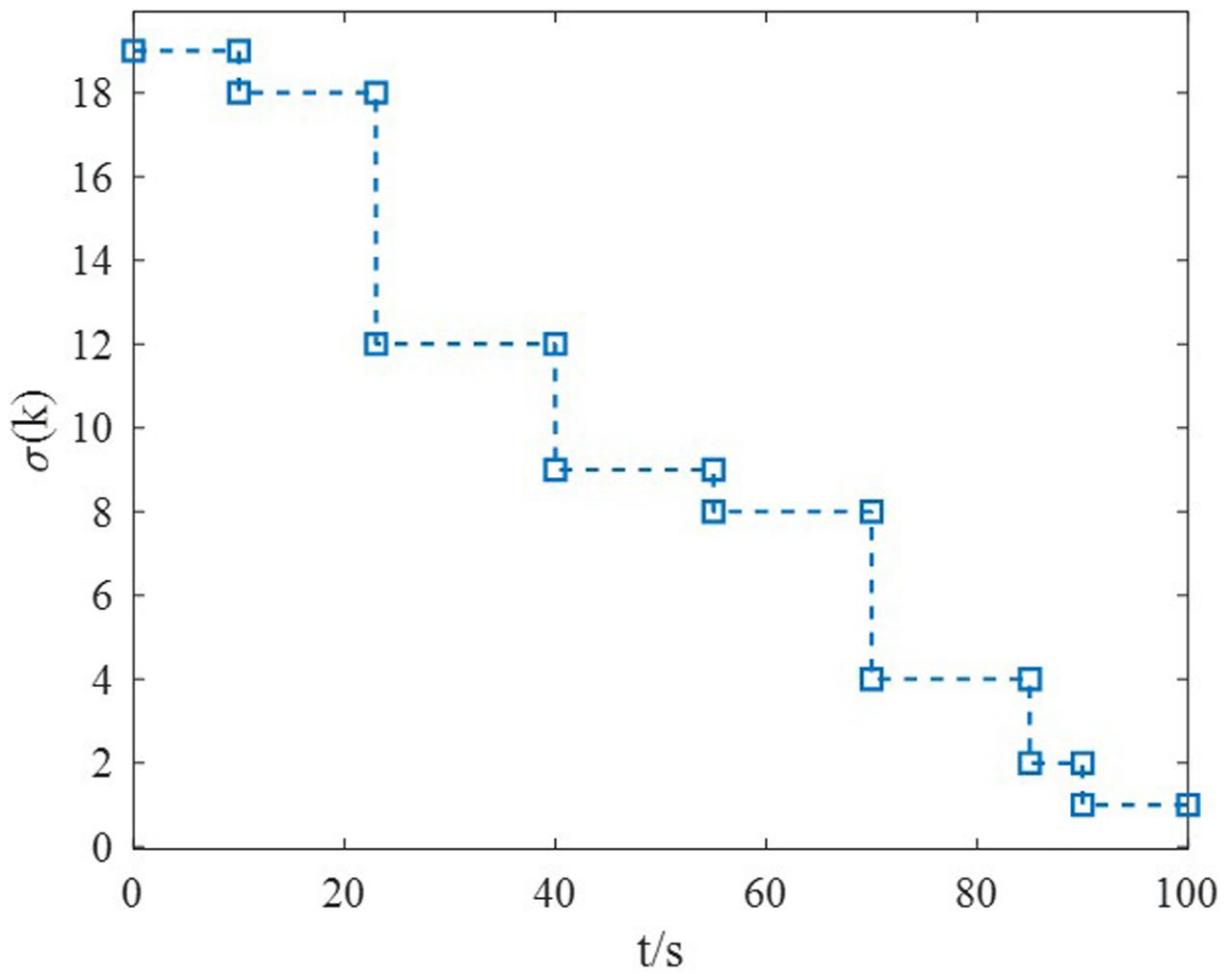
Switching logic of HiMAT in the flight envelope.

The switching of subsystems in the flight envelope is supposed to be 19-18-12-9-8-4-2-1, which is described in Figure 2.

The harmonics wind gust is considered in the paper, which is described in Eq. (83).


(83) $$\left\{ \begin{array}{l} p\left(k+1\right)=\left[\begin{array}{cc} 0.9922 & 0.1247\\ -0.1247 & 0.9922 \end{array}\right]p\left(k\right)\\ d\left(k\right)=\left[\begin{array}{cc} 1 & 0 \end{array}\right]p\left(k\right) \end{array}\right.$$

where $p\left(k\right)$ represents the state of external disturbance with initial value of $\left[0.01;0\right]$ .Furthermore, a command filter was provided to improve the performance of the intelligent tracking controller, which can be generated as follows:


(84) $$\left\{ \begin{array}{l} \begin{array}{c} J\left(k+1\right)=\left[\begin{array}{l} J_{1}\left(k+1\right)\\ J_{2}\left(k+1\right) \end{array}\right]\\ =\left[\begin{array}{c} J_{2}\left(k\right)\\ 2\zeta_{n}\omega_{n}\left\{S_{v}\left[\frac{\omega_{n}^{2}}{2\zeta_{n}\omega_{n}}\left(S_{a}-J_{1}\left(k\right)\right)\right]-J_{2}\left(k\right)\right\} \end{array}\right] \end{array}\\ z\left(k\right)=\left[\begin{array}{l} z_{1}\left(k\right)\\ z_{2}\left(k\right) \end{array}\right]=\left[\begin{array}{c} J_{1}\left(k\right)\\ J_{2}\left(k\right) \end{array}\right] \end{array}\right.$$

where $J\left(k\right)$ denotes the state vector; $z\left(k\right)$ represents the output of the filter; $\zeta_{n}$ and $\omega_{n}$ are the damping ratio and band width; $S_{a}$ and $S_{v}$ denote the transfer functions of the amplitude limiting and the rate limiting filters.

The parameters of the switched systems are given as $c_{1}=0$ , $c_{2}=1.5$ , $N_{f}=25$ , $\bar{\omega }=5$ , and R=I . Compared with the conventional ADT method, tighter bounds on FTS analysis can be obtained. The ADT method can be considered to be a special case of the MDADT method, and we can obtain that $\tau_{ai}^{*}\leq \tau_{a}^{*}$ , which is illustrated in Table 1. Therefore, the proposed method can realize limited conservative results than the ADT method. We set the probability of data missing as ρ=0.95 , the maximum number of consecutive data missing *N*_1_ is set to be 5. Moreover, the matrices $U_{1i}$ , $U_{2i}$ , $S_{1i}$ , and $S_{2i}$ can be solved by Eqs. (62), (63) in Theorem 3. The dynamics-based controller was constructed, and its parameter matrices and structure are given as follows:


(85) $$\begin{array}{l} K_{1}=\left[\begin{array}{ccc} 138.4164 & 2.4407 & -6.7702\\ 167.8987 & 0.9985 & -7.5232\\ 383.8513 & -4.9224 & -17.4799 \end{array}\right],K_{2}=\left[\begin{array}{ccc} 101.5134 & 1.6037 & -3.8630\\ 119.6668 & 0.8277 & -4.3093\\ 276.3462 & -1.8812 & -10.0604 \end{array}\right],\\ K_{4}=\left[\begin{array}{ccc} 96.5387 & 1.2329 & -2.9000\\ 93.0939 & 0.8237 & -2.1852\\ 268.1340 & -0.7194 & -7.2606 \end{array}\right],K_{8}=\left[\begin{array}{ccc} 175.2877 & 1.6404 & -6.3880\\ 61.4511 & 1.1225 & -2.3991\\ 391.6398 & -0.1709 & -14.2824 \end{array}\right],\\ K_{9}=\left[\begin{array}{ccc} 143.3570 & 1.4171 & -6.7477\\ 71.8536 & 0.9124 & -3.4568\\ 359.6986 & 0.5128 & -16.8429 \end{array}\right],K_{12}=\left[\begin{array}{ccc} 135.7652 & 1.2744 & -7.2143\\ 84.5744 & 0.9719 & -5.9774\\ 399.1680 & 1.8255 & -23.1364 \end{array}\right],\\ K_{18}=\left[\begin{array}{ccc} 221.0958 & 4.4685 & -11.2652\\ 162.9903 & 3.1023 & -8.3565\\ 555.0112 & 7.9902 & -29.2663 \end{array}\right],K_{19}=\left[\begin{array}{ccc} 251.0396 & 5.1211 & -13.9648\\ 113.9665 & 2.3356 & -5.9639\\ 762.3699 & 11.0262 & -40.3752 \end{array}\right] \end{array}$$

**Table 1:** Dwell time of various switching logics.

Switching logic	Parameter	Result
MDADT	$a_{1}=0.22$ , $a_{2}=0.24$ , $a_{4}=0.23$ , $a_{8}=0.19$ , $a_{9}=0.31$ , $a_{12}=0.26$ , $a_{18}=0.27$ , $a_{19}=0.28$ , $b_{1}=0.03$ , $b_{2}=0.04$ , $b_{4}=0.02$ , $b_{8}=0.05$ , $b_{9}=0.03$ , $b_{12}=0.04$ , $b_{18}=0.03$ , $b_{19}=0.03$ , $\Delta_{1}=2$ , $\Delta_{2}=1$ , $\Delta_{4}=3$ , $\Delta_{8}=3$ , $\Delta_{9}=2$ , $\Delta_{12}=1$ , $\Delta_{18}=3$ , $\Delta_{19}=1$ , $\mu_{1}=1.15$ , $\mu_{2}=1.21$ , $\mu_{4}=1.30$ , $\mu_{8}=1.11$ , $\mu_{9}=1.12$ , $\mu_{12}=1.22$ , $\mu_{18}=1.13$ , $\mu_{19}=1.25$ .	$\tau_{a1}^{*}=7.7101,\tau_{a2}^{*}=4.3390$ $\tau_{a4}^{*}=10.7211,\tau_{a8}^{*}=16.8154$ $\tau_{a9}^{*}=4.2968,\tau_{a12}^{*}=3.7734$ $\tau_{a18}^{*}=7.3806,\tau_{a19}^{*}=3.4131$
ADT	a=0.19 , b=0.05 , Δ=3 , μ=1.11	$\tau_{a}^{*}=16.8154$

Moreover, to overcome the problem of operation points with static instability, an angular rate compensator was introduced as follows:


(86) $$T_{f}\left(s\right)=\frac{k_{q}\left(s+1/t_{q}\right)}{s}$$

where $T_{f}\left(s\right)$ denotes the transfer function of angular rate compensator, $t_{q}$ and $k_{q}$ are the parameters of compensator.

Next, we presented two examples to validate the proposed method.

**Figure 3:**
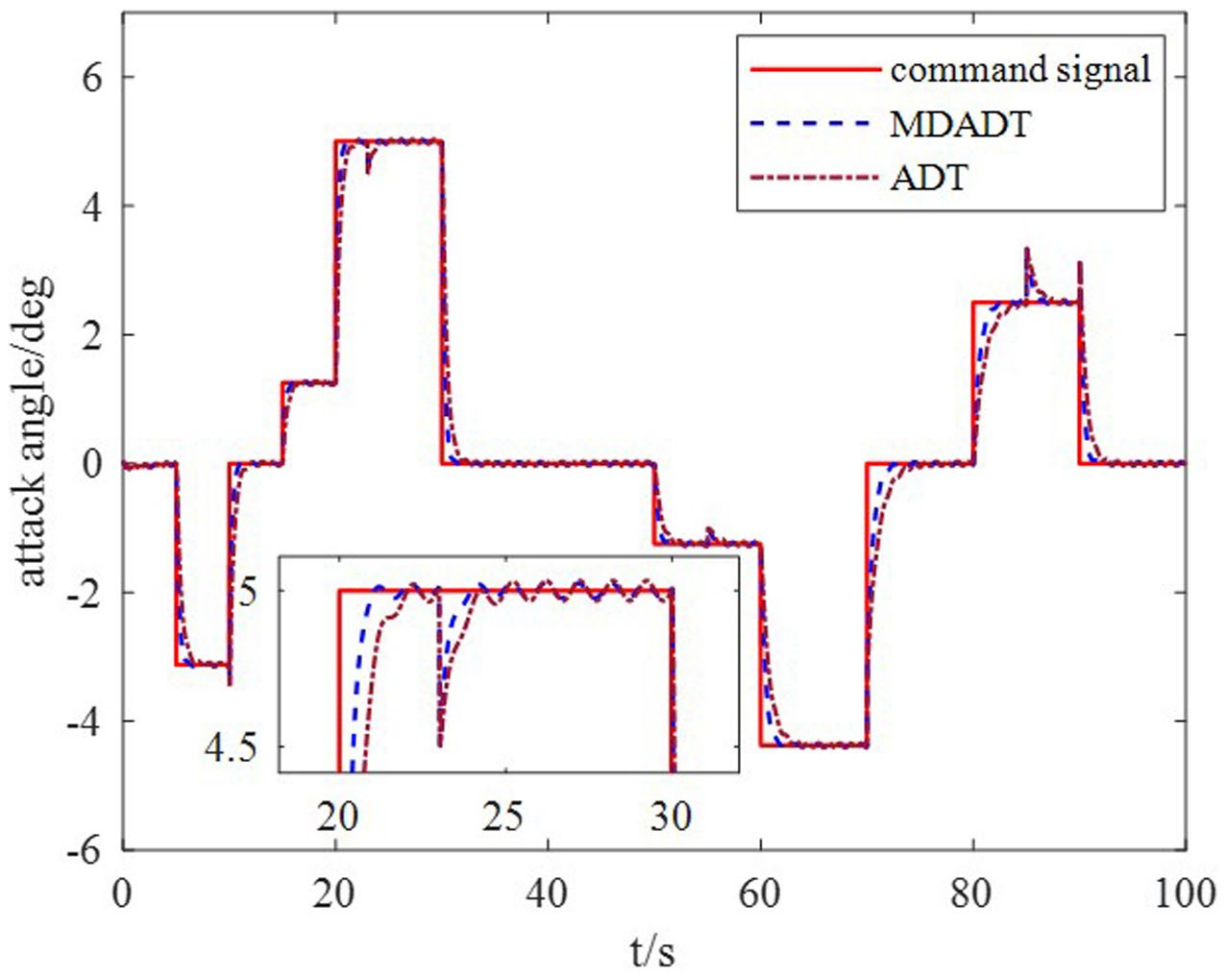
Response of the attack angle.

**Figure 4:**
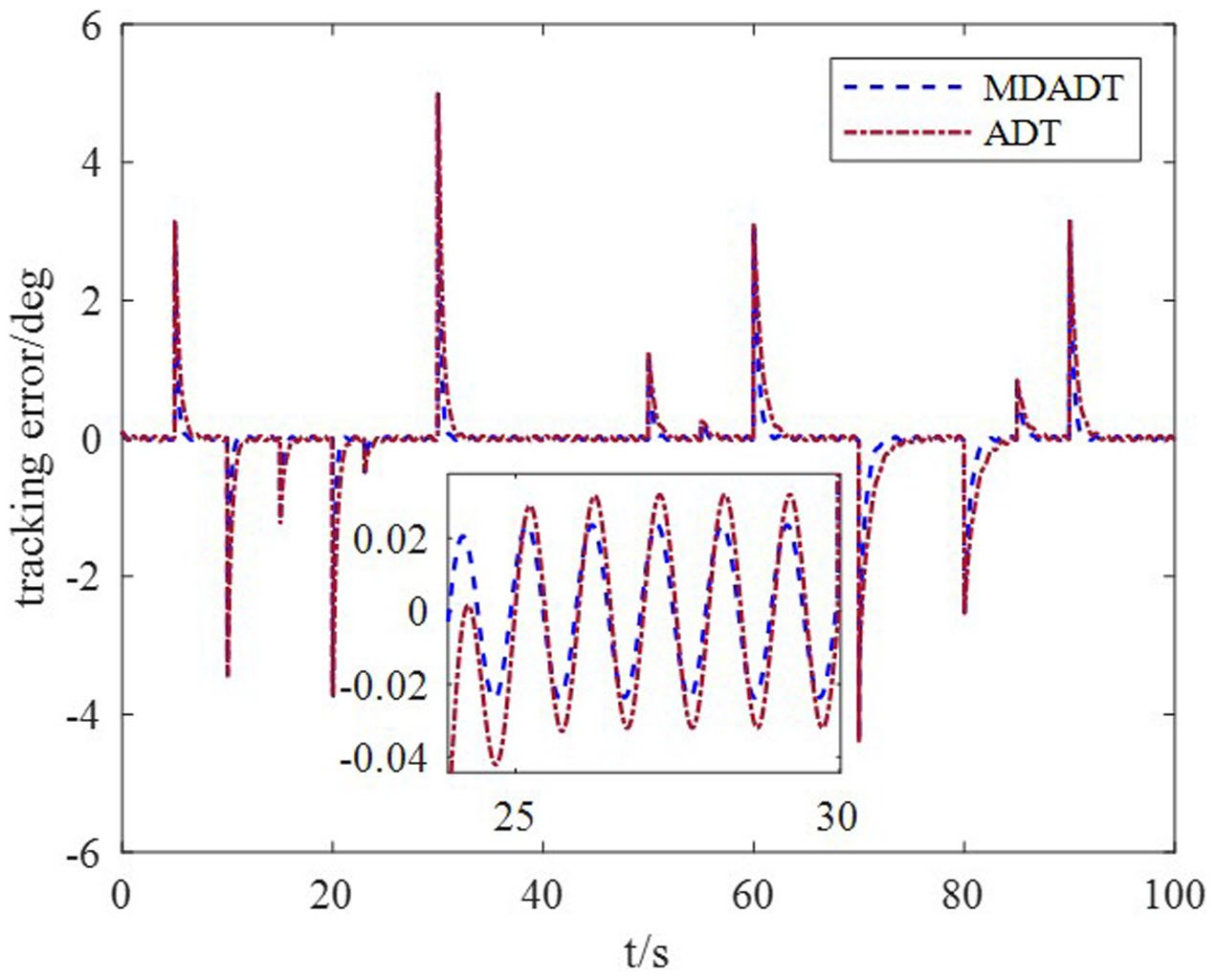
Tracking error.

*Example 1*: The tighter bounds on the dwell time can be obtained by the proposed method according to the data in Table 1. Moreover, because the characteristic of each subsystem is considered, the transient performance can be achieved by using the MDADT method. The switching of subsystems is displayed in Figure 2. Notably, the parameters of flight vehicles switch at the switching instants. First, to compare the difference between the two switching logic mechanisms, the simulation results under ADT switching logic and MDADT switching logic are displayed in Figures 3, 4, in which the labels are defined as ADT and MDADT, respectively. Figures 3, 4 reveal that the curves of the attack angle highlight the tracking performance in the flight envelope of switched controllers under ADT switching logic and MDADT switching logic. Thus, the tracking error can converge within the given time interval, and the transient performance of MDADT method is superior. Moreover, in Figures 3, 4, we provide the detailed enlargement of simulation curves near the switching time and steady process. Switched controllers with MDADT logic can achieve better transient performance than the those of controllers with ADT logic. Furthermore, the MDADT method corresponds to smoother response. The switched controllers with MDADT logic can obtain excellent transient performance with tighter bounds on the dwell time, which is less conservative than the ADT logic.

Example 2. In this section, the feasibility of the ADDPG algorithm for flight aircraft is validated. The weights of actor network and critic network are updated such that the learning-based controller adaptively compensates the model uncertainties and external disturbance in the environment. The action of supplementary control is added to the dynamics-based controller, which constitutes the real-time finite-time adaptive tracking control for the flight vehicles. The design parameters of the ADDPG algorithm are defined in Table 2.

**Table 2:** Parameters setting of the ADDPG.

Parameter	Value
Discount factor	0.9
Learning rate of the critic network	0.0005
Learning rate of the actor network	0.0005
Mini-batch size	32
Replay buffer size	1,000
Length of each segment	100
Activation function	ReLu
[*v*_1min_, *v*_1max_]	[−3, 3]
[*v*_2min_, *v*_2max_]	[−10, 2]
*l*_1min_	10^−6^
*l*_2min_	10^−6^

The input is divided into two paths for critic networks, corresponding to the observation and action. The number of neurons in the input layer of the observation path is the dimension of the observed states, which is represented by *obs*. The number of neurons in the input layer of the action path corresponding to the controller parameters. The critic networks are updated based on the adaptive moment estimation (Adam) algorithm. The regularization factor is set to be $2\times 10^{-4}$ .

We define the input of actor network is the observed states and the output is the compensated controller parameters. The activation function of fully connected layers is set to be ReLu and the activation function of output layer is tanh. The weights of actor network are updated based on the Adam algorithm. The variance of noise is set to be 0.1 and the variance decay rate is $1\times 10^{-5}$ . Because the stability and robustness of the closed-loop system are guaranteed by the switched control theory and robust control theory, we consider wind gust in the training environment, the perturbations of aerodynamic parameters and wind gust are introduced in the testing environment. Then the algorithms can be implemented on a desktop with Intel Core i7-10700K @3.80GHz RAM 16.00 GB and operation system of Windows 10.

**Figure 5:**
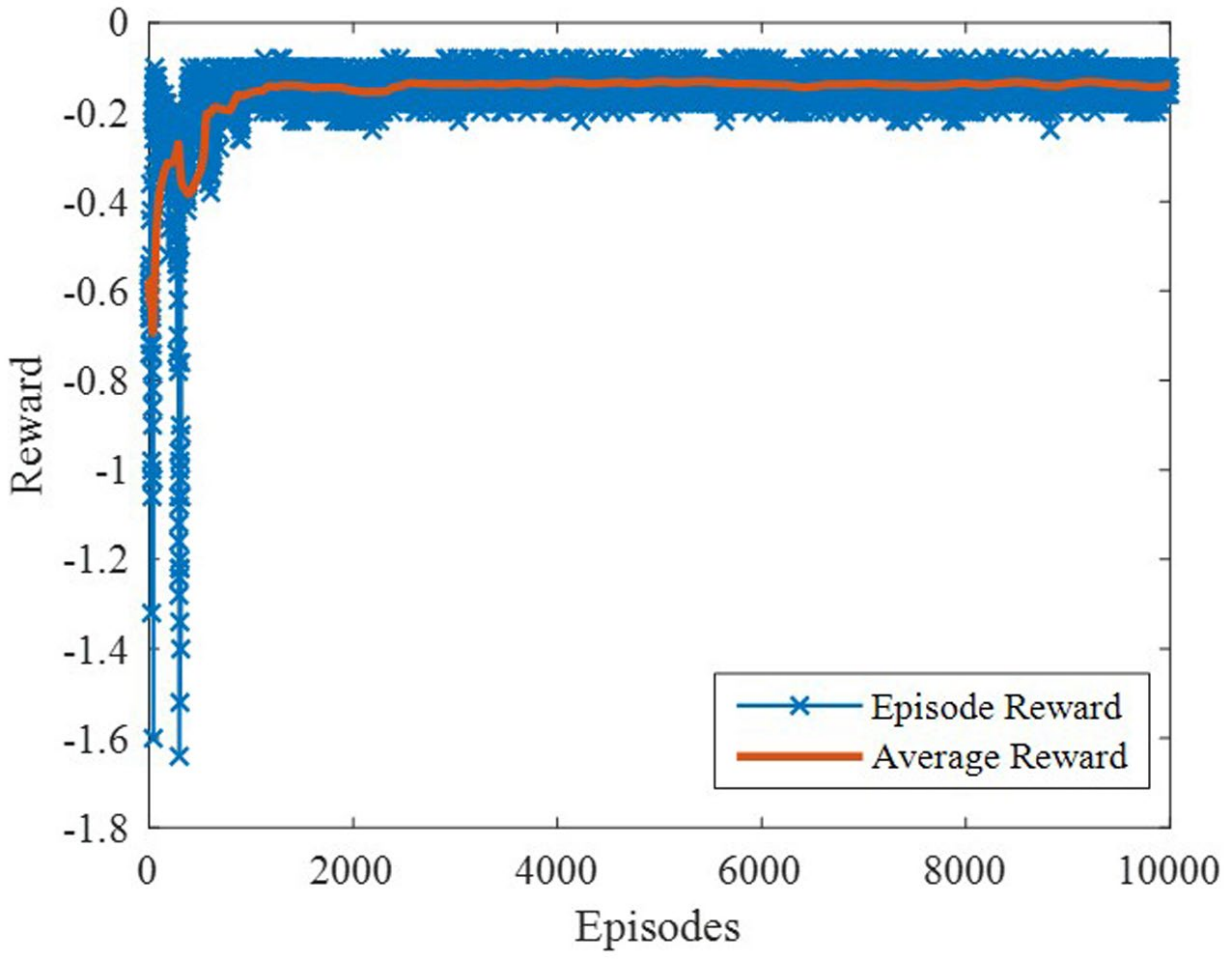
Episodes reward of the ADDPG.

**Figure 6:**
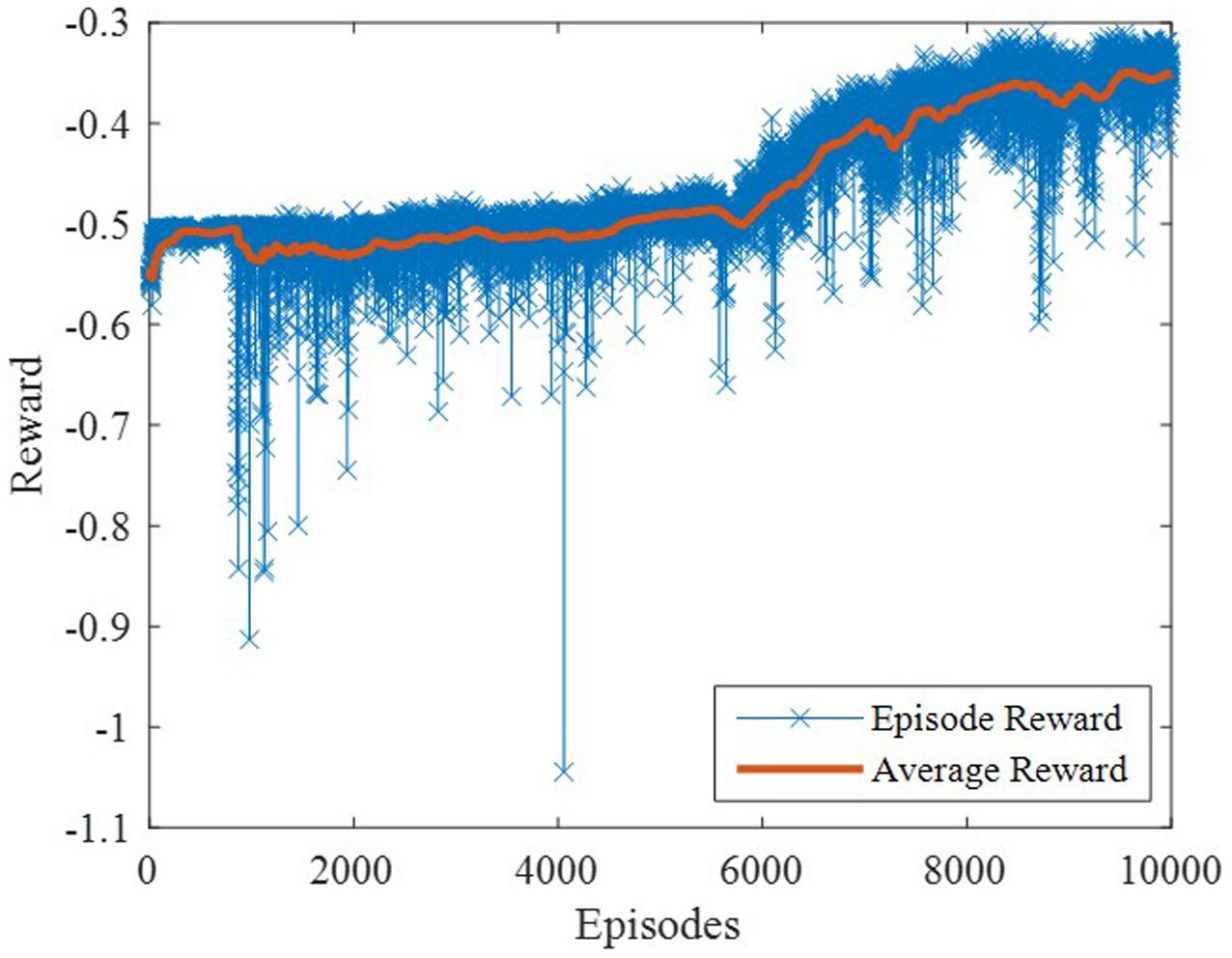
Episodes reward of the DDPG.

**Figure 7:**
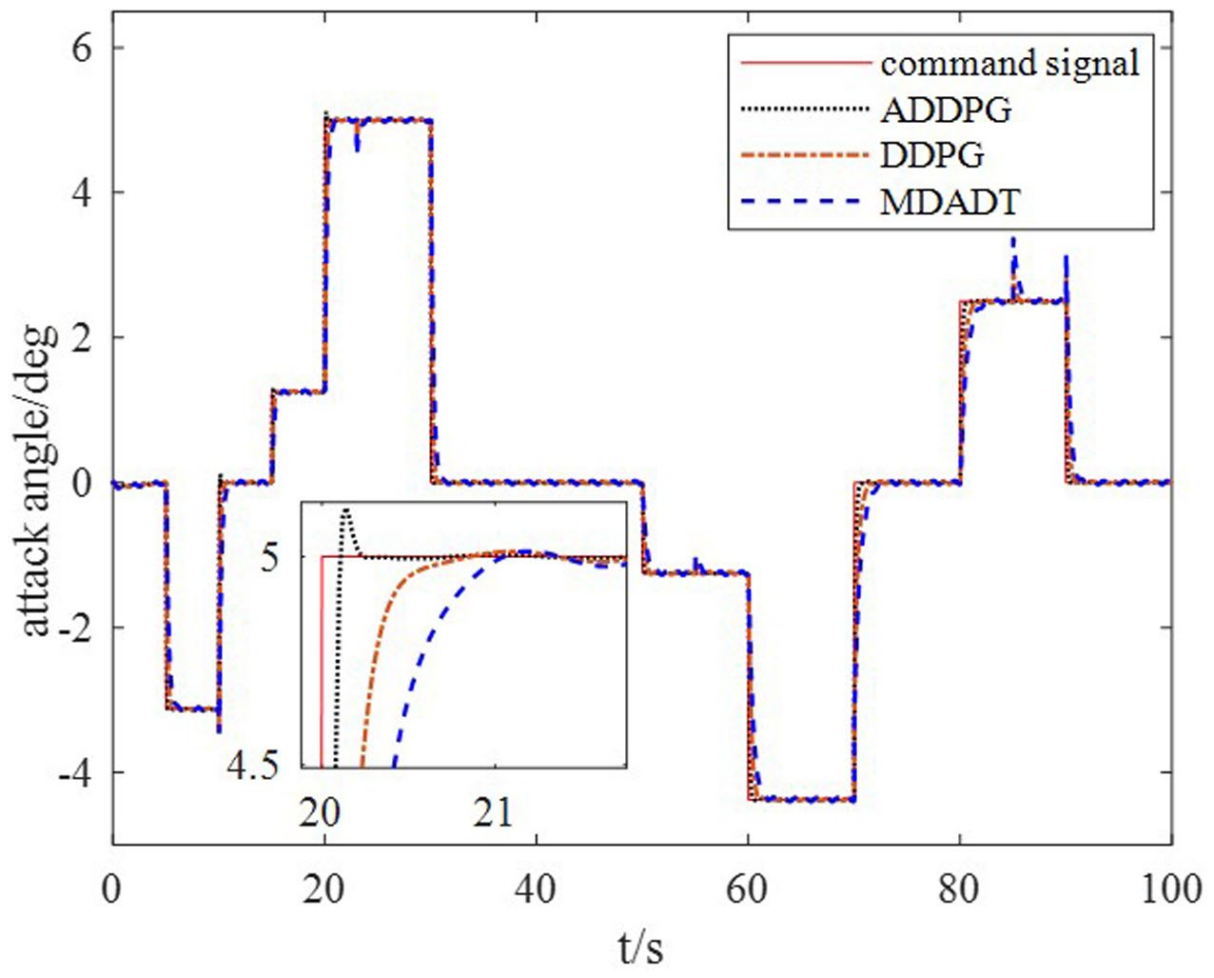
Response of the attack angle.

**Figure 8:**
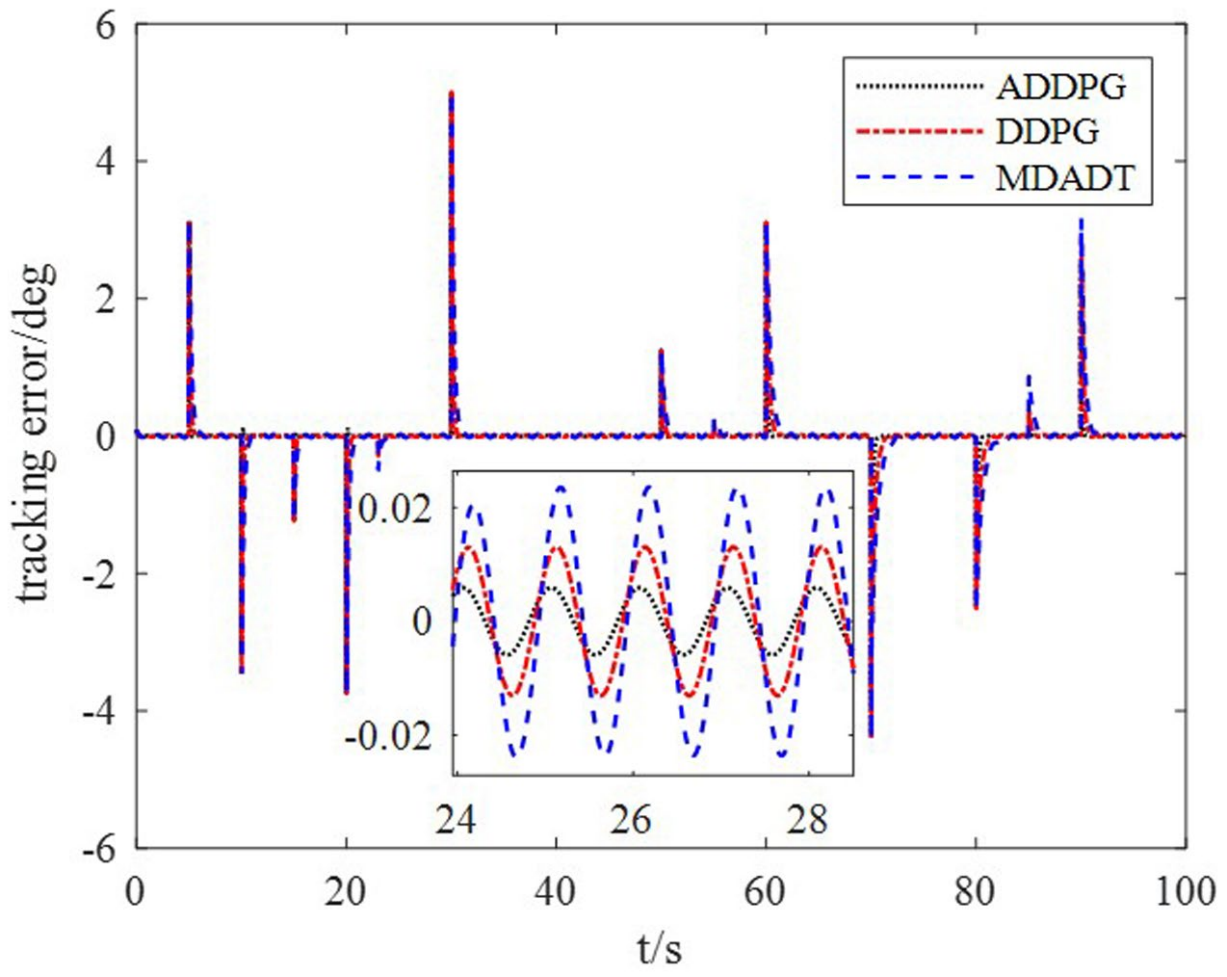
Tracking error.

**Figure 9:**
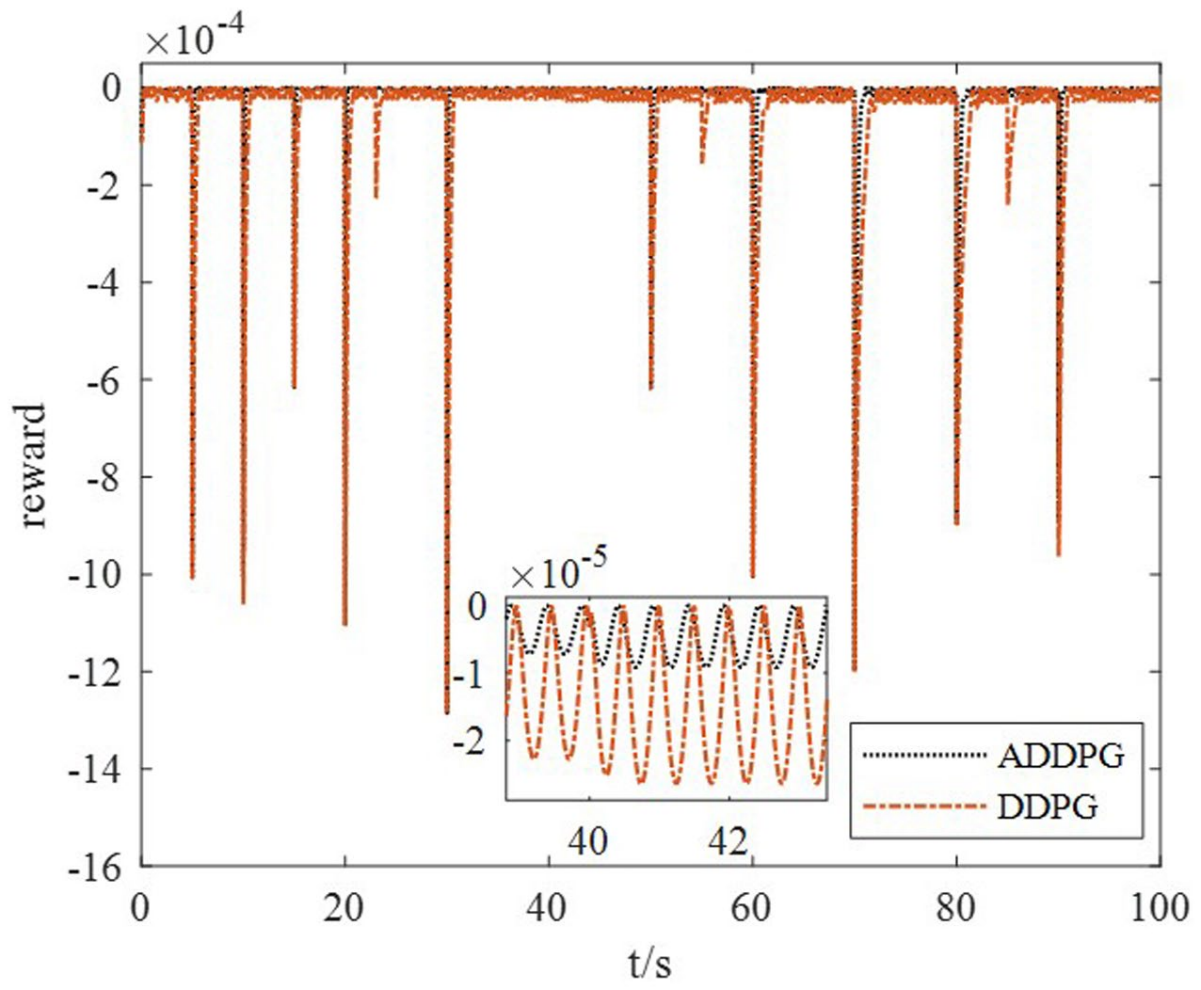
Response of reward function.

The DDPG algorithm was simulated to verify the advantages of the proposed method in terms of control performance and convergence for algorithms. The robust controller proposed by the MDADT method was designed as the dynamics-based controller. Both the ADDPG and DDPG algorithms are given in the simulation as the learning-based controller to compensate the unexpected uncertainties in the flight environment. The simulation results are displayed in Figures 5–9, in which the MDADT method, MDADT with DDPG method, and MDADT with ADDPG method are labeled as MDADT, DDPG, and ADDPG, respectively. As displayed in Figures 5, 6, the ADDPG algorithm outperformed the episodes reward convergence of DDPG algorithm, which required fewer episodes to converge in the neighbor of the origin. Therefore, the ADDPG algorithm outperformed the conventional DDPG algorithm in terms of the control performance and steady error. The responses of attack angle are displayed in Figure 7. Both DDPG and ADDPG algorithms could achieve convergence and efficient performance. However, the transient convergence of the ADDPG algorithm was superior to that of the DDPG algorithm. The tracking errors are displayed in Figure 8. The controller compensated with the DDPG and ADDPG algorithms can exhibit improved performance of steady-state response. However, the steady-state error of the ADDPG algorithm was less than that of the DDPG algorithm. The reward function of an episode is displayed in Figure 9. The ADDPG algorithm can achieve superior final performance.

The average tracking errors of methods are presented in Table 3. The online scheduling through DDPG and ADDPG can efficiently reduce the average tracking error; the adaptive reward function can improve the tracking performance. The proposed method can overcome the undesirable response caused by asynchronous switching and uncertainties in the flight environment.

**Table 3:** Average tracking errors.

Methods	Value (deg)
ADT	0.2053
MDADT	0.1196
DDPG with MDADT	0.0711
ADDPG with MDADT	0.0326

**Figure 10:**
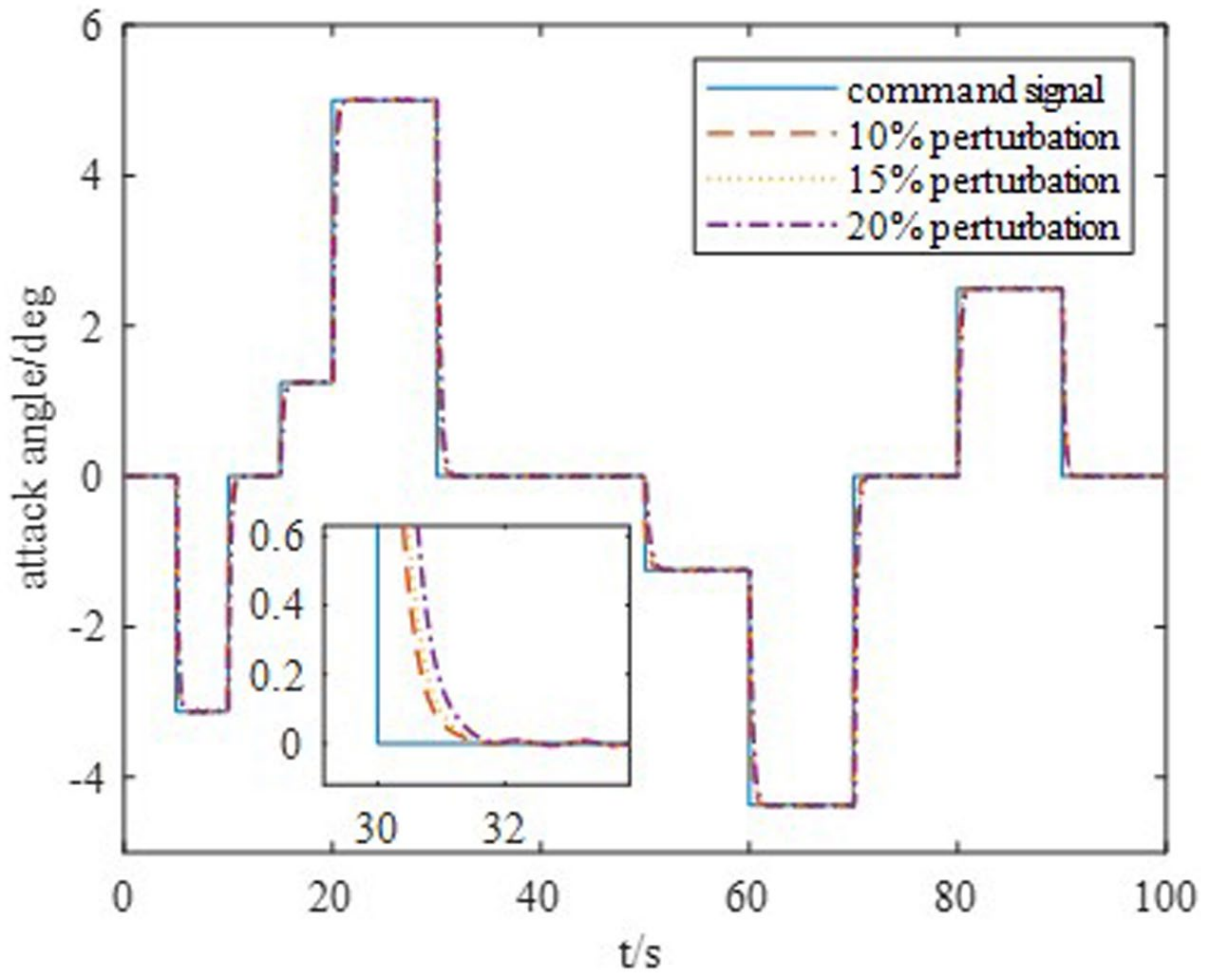
Response of the attack angle.

**Figure 11:**
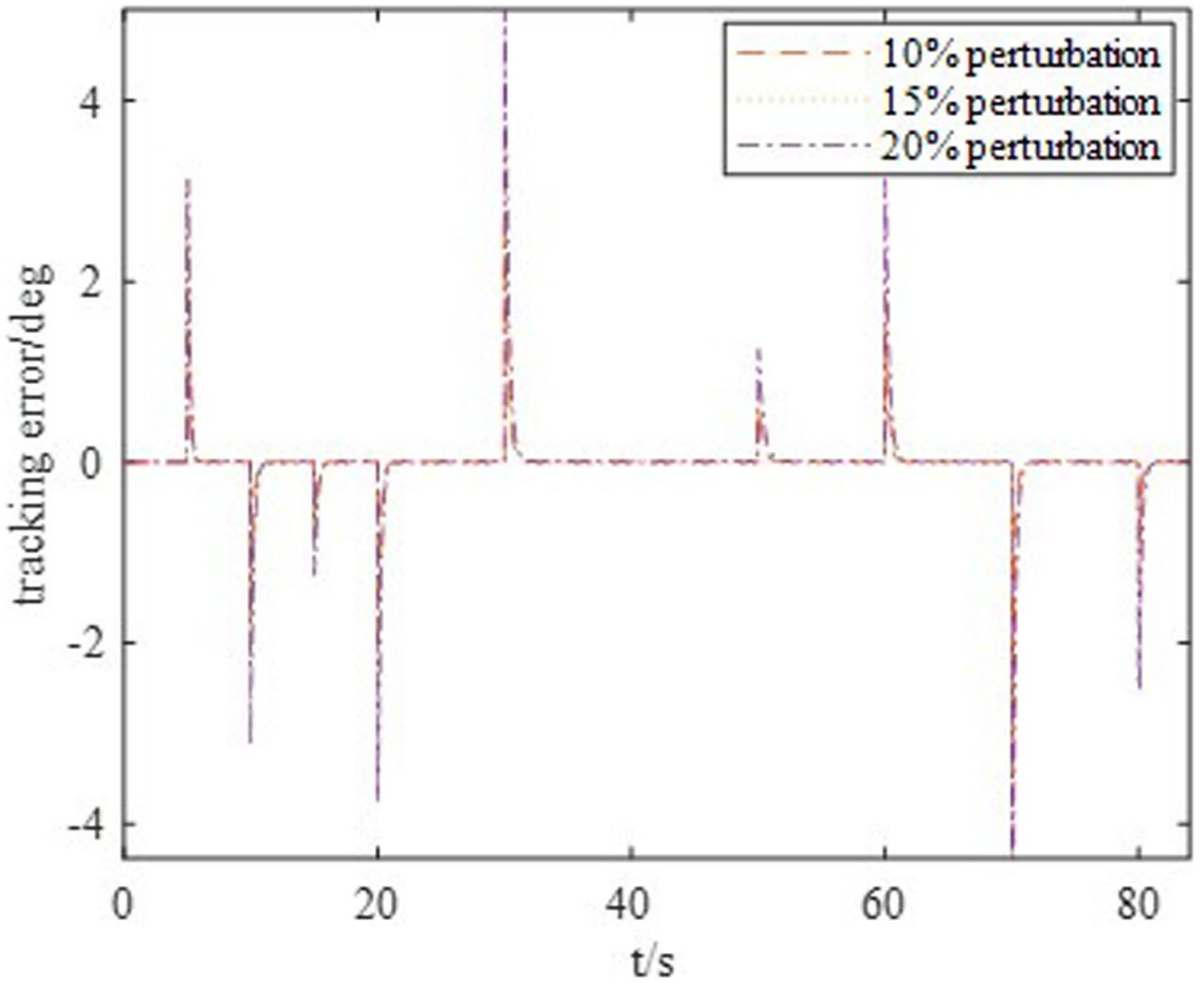
Tracking error.

Moreover, to show the effectiveness to deal with system uncertainties and disturbance, we give the simulation results of HiMAT vehicle with disturbances and uncertainties of aerodynamic parameters, which can also illustrate the potential application prospects for practical environment. The results are described in Figures 10, 11, in which we consider the cases where the aerodynamic parameter perturbations are 10, 15, and 20%. The responses of attack angle are given in Figure 10 and the tracking errors are given in Figure 11. The average tracking errors in the presence of aerodynamic perturbations are also provided in Table 4. We can see that the stability and tracking performance can be guaranteed with uncertainties and disturbances by using the proposed method, which illustrates that the proposed method can ensure the control accuracy, stability, and robustness simultaneously.

**Table 4:** Average tracking errors in the presence of aerodynamic perturbations.

Methods	Value (deg)
10% perturbation	0.0442
15% perturbation	0.0576
20% perturbation	0.0898

*Remark 4*: We draw inspiration from the traditional method of dealing with the sim-to-real transfer issue. Firstly, the nonlinear model is converted to a linear model by employing Jacobian linearization. Then we can design the nominal controller on the reference points. In most engineering applications, the stability margin is introduced and analyzed to ensure the robustness. Similarly, in this paper, we developed finite-time robust control theory to ensure the stability and attenuation performance. The uncertainties and disturbances in practical environment can be overcome. However, we noticed that it is difficult to realize optimal compromise between robustness and transient performance. The ADDPG algorithm is given to improve the control accuracy. Moreover, the non-fragile control theory is introduced, which ensures the stability and prescribed attenuation performance on the scheduling intervals.

*Remark 5*: The problem of finite-time tracking control for switched flight vehicles was investigated. According to the numerical examples, the advantages of the suggested control method to address the flight vehicle considering disturbances and uncertainties over the existing control methods are demonstrated, which can be described as follows: (1) Unlike the conventional model-based control methods, the proposed method was developed by using DRL, which can improve control performance and overcome the undesirable response caused by uncertainties. (2) In the proposed method, the advantages of model-based and model-free method are combined. The dynamics-based controller was developed to ensure stability and robustness, and the learning-based controller was proposed to compensate the uncertainties in the flight environment. (3) The established adaptive generalized reward function can improve convergence and robustness.

## 5 Conclusion

The finite-time control of switched flight vehicles with asynchronous switching was realized using a novel nonfragile DRL method. The flight vehicles were modeled as the switched system, and the asynchronous switching caused by packet dropouts was considered. The MDADT and MLF methods were used to ensure FTS and weighted prescribed attenuation index. LMIs were used to determine the solutions of the finite-time tracking controller. To compensate the external disturbance and improve tracking performance, the ADDPG algorithm based on the actor–critic framework was provided to optimize the parameters of tracking controllers. To improve optimization efficiency and decrease computational complexity, parameter optimization was assumed to be limited in the given range. The compensation of control policy in a given range is considered as the uncertainties of the controller parameters, and the FTS is ensured by nonfragile control theory. Compared with the conventional DDPG algorithm, the adaptive hyper parameters of reward function were introduced to achieve superior control performance and realize a general case. The FTS, robustness, and transient performance were ensured simultaneously by the proposed method. In the future, the following four points should be studied: (1) The event-triggered control structure should be considered to reduce the load and improve the robustness of information transformation. (2) The parallel optimization methods should be presented to improve training efficiency. (3) The fitting ability and generalization ability of neural networks should be studied to improve the robustness in the complex environment. (4) The semi physical simulations and flight tests of mini drones should be developed to further demonstrate the engineering feasibility of the proposed method.

## Data availability statement

The original contributions presented in the study are included in the article/supplementary material, further inquiries can be directed to the corresponding author.

## Author contributions

HC: Writing – original draft, Writing – review & editing. RS: Writing – original draft, Writing – review & editing. HL: Writing – review & editing, Writing – original draft. WW: Writing – review & editing, Writing – original draft. BZ: Writing – review & editing, Writing – original draft. YF: Writing – original draft, Writing – review & editing.
